# The Honey Volatile Code: A Collective Study and Extended Version

**DOI:** 10.3390/foods8100508

**Published:** 2019-10-17

**Authors:** Ioannis K. Karabagias, Vassilios K. Karabagias, Anastasia V. Badeka

**Affiliations:** Laboratory of Food Chemistry, Department of Chemistry, University of Ioannina, 45110 Ioannina, Greece; vkarambagias@gmail.com (V.K.K.); abadeka@uoi.gr (A.V.B.)

**Keywords:** honey variety, honey code, HS-SPME/GC-MS, data handling, data bank, chemometrics

## Abstract

Background: The present study comprises the second part of a new theory related to honey authentication based on the implementation of the honey code and the use of chemometrics. Methods: One hundred and fifty-one honey samples of seven different botanical origins (chestnut, citrus, clover, eucalyptus, fir, pine, and thyme) and from five different countries (Egypt, Greece, Morocco, Portugal, and Spain) were subjected to analysis of mass spectrometry (GC-MS) in combination with headspace solid-phase microextraction (HS-SPME). Results: Results showed that 94 volatile compounds were identified and then semi-quantified. The most dominant classes of compounds were acids, alcohols, aldehydes, esters, ethers, phenolic volatiles, terpenoids, norisoprenoids, and hydrocarbons. The application of classification and dimension reduction statistical techniques to semi-quantified data of volatiles showed that honey samples could be distinguished effectively according to both botanical origin and the honey code (*p* < 0.05), with the use of hexanoic acid ethyl ester, heptanoic acid ethyl ester, octanoic acid ethyl ester, nonanoic acid ethyl ester, decanoic acid ethyl ester, dodecanoic acid ethyl ester, tetradecanoic acid ethyl ester, hexadecanoic acid ethyl ester, octanal, nonanal, decanal, lilac aldehyde C (isomer III), lilac aldehyde D (isomer IV), benzeneacetaldehyde, *alpha*-isophorone, 4-ketoisophorone, 2-hydroxyisophorone, geranyl acetone, 6-methyl-5-hepten-2-one, 1-(2-furanyl)-ethanone, octanol, decanol, nonanoic acid, pentanoic acid, 5-methyl-2-phenyl-hexenal, benzeneacetonitrile, nonane, and 5-methyl-4-nonene. Conclusions: New amendments in honey authentication and data handling procedures based on hierarchical classification strategies (HCSs) are exhaustively documented in the present study, supporting and flourishing the state of the art.

## 1. Introduction

The high consumer demand for authentic products along with the pressure on the market with products of low quality, distributed by cheap producing countries, creates the need for a multi-optional handling of natural-based products. A typical example of such products comprises honey—the sweet viscous solution obtained through the action of honeybees (*Apis mellifera*). The main types of honey include nectar and honeydew honeys. Nectar honeys are produced via the collection of the nectar of flowers by the honeybees.

On the other hand, honeydew honeys are characterized by the presence of secretions of plant-sucking insects (Hemiptera) living in the parts of the plants or conifer trees [1]. Given the historical meaning and symbolism of honey through the welfare of many civilizations [2], the latter has been subjected to exhaustive research. Apart from the basic components which are sugars and moisture, there are plenty of micro-constituents including minerals, phenolic compounds, organic acids, proteins, free amino acids, vitamins and volatile compounds and traces of lipid acids that have attracted researchers [3,4,5,6,7]. 

Among the aforementioned micro-constituents, volatile compounds are considered among the key parameters of honey sensory attributes. These contribute to the aroma providing the consumers with the emotional feelings related to regular consumption. It has been reported in the literature that volatile compounds of honey number in the hundreds, including esters, ethers, alcohols, acids, aldehydes, hydrocarbons, ketones, terpenes, norisoprenoids, carotenoid derivatives, furan and pyran derivatives, phenolic volatiles, benzene derivatives, quinones and other biomolecules originating from plants or bacteria metabolism with potential applications. The presence and quantity of these volatile compounds depends on the botanical and geographical origin of the honey [6,7,8]. 

The application of instrumental techniques has greatly favored the identification of the volatile compounds of honey. Numerous studies have been published using hydrodistillation, liquid–liquid extraction, simultaneous steam distillation extraction or Likens–Nickerson simultaneous distillation extraction micro-simultaneous steam distillation–solvent extraction, and ultrasonic solvent extraction for this purpose [7]. Some key volatile compounds that have been reported in the literature include benzene derivatives and phenolic volatiles for the case of strawberry tree honey [9]. Nonanal and *cis*-linalool oxide [2-[(*2S,5R*)-5-ethenyl-5-methyloxolan-2-yl]propan-2-ol] in combination with benzene derivatives and phenolic volatiles for Italian and Greek chestnut honeys [6,10]. Benzaldehyde, benzeneacetaldehyde and phenylethylalcohol were reported to be some characteristic volatile compounds of Spanish citrus and honeydew honeys [11,12]. The key volatile compounds of pine, fir, citrus, thyme, honeydew, and flower honeys harvested in Italy, Spain, Turkey, Greece, Morocco, and Brazil include benzaldehyde, benzeneacetaldehyde, octanal, nonanal, decanal, and different isomers of lilac aldehyde [3,4,6,8,13,14,15,16]. Norisoprenoids such as isophorone and 4-ketoisophorone (2,6,6-trimethyl-2-cyclohexene-1,4-dione) have been previously reported to serve as volatile markers of the floral origin of Sardinian strawberry tree and Indian saffron honeys [5,9]. *Alpha*-pinene, terpinolene, 2-phenylacetate and numerous other volatile compounds were considered as markers of the provenance of Argentinean honeys [17]. 

Based on the aforementioned, the objectives of the present study, which is collective in nature, were: (a) to classify clover, citrus, chestnut, eucalyptus, fir, pine, and thyme honeys from different countries (Egypt, Greece, Morocco, Spain, and Portugal) according to botanical origin based on the use of specific volatile compounds in combination with chemometrics and (b) classify honey samples according to the honey code—that is, a combination of the grammatical sequences of the different honey types used in the study by using the first letter of each honey type nomenclature. To the best of our knowledge, over the last 10 years, this is only the second study in the literature that implements, among others, a hierarchical classification strategy (HCS) for honey authentication [10], and the novelty of the study herein is highlighted by the use of a large number of different types of honey samples harvested in different parts of the world. Therefore, the whole procedure is more complicated and exhaustive “crash tests” are provided with the use of a multivariate analysis of variance (MANOVA), linear discriminant analysis (LDA), *k*-nearest neighbors (*k*-NN), and factor analysis (FA).

## 2. Materials and Methods

### 2.1. Honey Samples

One hundred and fifty-one honey samples (*n* = 151) were collected between the years 2011 and 2018 from Egypt, Greece, Morocco, Spain, and Portugal. Honey samples from Greece were obtained from Attiki Bee Culturing Co. Alex.Pittas S.A. (Athens, Greece); honey samples from Egypt, Spain and Morocco were obtained from local shops; honey samples from Portugal were obtained from APISMAIA (Povoa de Varzim, Portugal) The honey samples were subjected to volatile compound analysis according to botanical origin as clover (*Trifolium alexandrinum*), citrus (*Citrus* spp.), chestnut (*Castanea sativa*), eucalyptus (*Eucalyptus* spp.), fir (*Abies cephalonica*), Pine (*Pinus* spp.) and thyme (*Thymus capitatus* L. and *Thymus* spp.), which was confirmed by melissopalynological analysis [16]. In particular, clover honeys (*n* = 8) originated from Egypt; citrus honeys originated from Egypt (*n* = 7), Spain (*n* = 8), Morocco (*n* = 6), and Greece (*n* = 10); chestnut honeys originated from Greece (*n* = 1) and Portugal (*n* = 3); eucalyptus honeys (*n* = 4) originated from Portugal; fir honeys (*n* = 31) originated from Greece; pine honeys (*n* = 39) originated from Greece; thyme honeys (*n* = 42) originated from Egypt (*n* = 7), Greece (*n* = 12), Morocco (*n* = 6), and Spain (*n* = 10). Honey samples were shipped to the laboratory and maintained firstly at room temperature in paper boxes for sampling which was started at once. Sampling and analysis followed the sequence of honey type harvesting through the aforementioned years. The paper boxes were kept away from UV light. The quantity of honey samples left was stored at 4 ± 1 °C.

### 2.2. Honey Code Development

The honey code was used to construct the group of objects that would be subjected to statistical analysis using the first letter of each honey type. Clover, citrus and chestnut honeys (*n* = 42) were represented by CCC; eucalyptus honeys (*n* = 4) by E; fir honeys (*n* = 31) by F; pine honeys (*n* = 39) by P, and thyme honeys (*n* = 42) by T. The main purpose of this hierarchical procedure was to test the classification ability of honey samples from different countries based on the use of specific volatile compounds and chemometrics according to honey type lettering (use of the first letter) [10].

### 2.3. Analysis of Gas Chromatography–Mass Spectrometry in Combination with Headspace Solid-Phase Microextraction (HS-SPME/GC–MS)

The experimental strategy for the isolation and semi-quantification of honey volatile compounds along with HS-SPME/GC–MS equipment and analysis conditions are given in details in previous work [10]. The mass spectral library used for the identification of volatile compounds was Wiley 7 (2005) of the National Institute of Standards and Technology (NIST). Only the volatile compounds that had ≥80% similarity with those of the Wiley MS library were tentatively identified using the GC–MS spectra. Data were expressed as concentration (Canalyte, mg/kg) based on the ratio of peak areas of the isolated volatile compounds to that of the internal standard (benzophenone) multiplied by the final concentration of the internal standard, assuming a response factor (RF) equal to 1 for all the compounds. An additional method of identification was considered and included the calculation of Kovats indices using a mixture of *n*-alkanes (C8–C20) which was supplied by Supelco (Bellefonte, PA, USA). The standard mixture was dissolved in *n*-hexane. The retention time of the standards was determined according to the temperature-programmed run used in the analysis of honey samples. MS and Kovats indices data were compared to those found in the Wiley MS library. A solvent delay of 5 min was inserted in the program, in order to avoid the elution of ethanol, in which the internal standard was dissolved. Each sample was analyzed in duplicate and the results were averaged.

### 2.4. Statistical Analysis

Multivariate analysis of variance (MANOVA), linear discriminant analysis (LDA), *k*-nearest neighbors (*k*-NN), and factor analysis (FA) were applied to the semi-quantitative data of volatile compounds. The first part of the statistical analysis included the botanical origin differentiation of clover, citrus, chestnut, eucalyptus, fir, pine, and thyme honeys based on the use of the significant volatiles (*p <* 0.05) which served as the independent variables. The botanical origin served as the grouping variable consisted of 7 groups (clover, citrus, chestnut, eucalyptus, fir, pine, and thyme honeys). In the second part of the statistical analysis, honey samples were grouped according to the honey code. The expectation was to investigate whether classification results could be improved.

For the MANOVA analysis, the indices of the multivariate hypothesis such as Pillai’s Trace, Wilks’ Lambda, Hotelling’s Trace, and Roy’s Largest Root were computed to determine whether there was a multivariate effect of volatile compounds (*p* < 0.05) on the botanical origin or the honey code of honey. The size of the effect was further evaluated by consideration of partial eta squared values (*η*^2^). It should also be stressed that the lower the value of Wilks’ Lambda, the higher the differences between groups of objects. 

Considering only the significant volatiles, LDA was then applied to classify honey samples according to group membership based on the use of original and cross-validation methods. LDA provides linear discriminant functions originating from the combinations of the significant variables (all independent variables are entered together/simultaneously in the analysis) multiplied by the standardized canonical discriminant function coefficients plus a constant, characteristic for each discriminant function [18]. Moreover, the tolerance test was also computed in the analysis. Tolerance may be defined as the proportion of a variable’s variance not accounted for by other independent variables in the created discriminant function. It practically shows that a variable with very low tolerance contributes little information to the predictive model and may cause computational problems [19].

For the *k*-nearest neighbors analysis, the botanical origin of samples or the honey code served as the target parameter, while the significant volatiles (*p* < 0.05) served as the features. The number of the *k*-nearest neighbors was set by default equal to the higher number provided by the SPSS software—that is, 3–5. The classification ability of the constructed model was estimated by the application of training and holdout partitions. In the training sample, 70% of the cases were randomly assigned to partitions, while the rest of the cases were assigned to the holdout sample. The distances of an unknown object from all the members of the training set were calculated using the Euclidean distance in multi-dimensional space. The *k*-smallest distances between the unknown object and the training set sample were then identified. K is normally a small odd number, and the unknown variable is allocated to the class with the majority of these *k* distances [10]. For the performance of feature selection among the population of significant variables, *k*-NN analysis was run again using only the specified predictors that built the model (usually 3–5) in the previous step, in order to reduce the number of predictors to those having the lower error rate and *k* distance.

Afterwards, a data mining technique such as factor analysis (FA) was applied to the significant parameters in order to explain the total variance of the constructed model in the multidimensional space. At the same time, FA provides a reduction in the variables, in order to gain the most pronounced ones with the higher correlation and communalities of independent latent variables. The communalities indicate the common variance shared by factors with specific variables. A higher communality indicates that a larger amount of the variance in the variable has been extracted by the factor solution. For the most effective data collection during FA, communalities should be ≥0.4. The extraction method was principal component analysis (PCA). The rotation method used was Varimax with Keiser Normalization. The Varimax rotation is used in statistical analysis to simplify the expression of a particular sub-space in terms of just a few major items each. The actual coordinate system (practically constant) is the orthogonal basis that is being rotated to align with these coordinates. The sub-space can be defined with either PCA or FA. Varimax maximizes the sum of the variances of the squared loadings (squared correlations between variables and factors [20]. 

The accuracy and strength of factor analysis was supported further by the Kaiser–Meyer–Olkin (KMO) test, which comprises a measure of how well suited the data is for factor analysis. The acceptable value considered was that of KMO ≥ 0.50. In addition, the effectiveness and suitability of factor analysis was explored using Bartlett’s test of sphericity. This test highlights the hypothesis that the correlation matrix is an identity matrix, which would indicate that the variables incorporated into the model are unrelated and therefore unsuitable for structure detection. Small probability values (*p* < 0.05) indicate that a factor analysis may be useful with data treatment [20]. Statistical analysis was run using the SPSS (version 20.0, IBM, Armonk, NY, USA) statistics software.

## 3. Results and Discussion

### 3.1. Volatile Compounds of Clover, Citrus, Chestnut, Eucalyptus, Fir, Pine, and Thyme Honeys

A considerable number of volatile compounds were putatively identified and semi-quantified. In total, 94 volatile compounds of different classes were isolated. Table 1 lists the volatile compounds according to retention time and their class. The majority of volatile compounds (62 volatiles) varied significantly (*p* < 0.05) according to the botanical origin of honey. 

**Figure 1:**
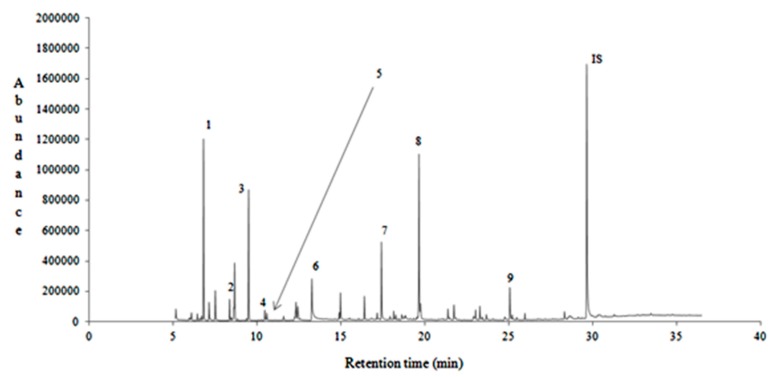
A typical gas chromatogram of clover honey (no. 3) from Egypt indicating selected key volatile compounds. 1: 2-methyl-Butanal. 2: 3-methyl-Butanal. 3: Heptane. 4: 3-methyl-1-Butanol. 5: 2-methyl-1-Butanol. 6: Furfural. 7: Octanal. 8: Nonanal. 9: Decanoic acid ethyl ester. IS: Internal standard.

Figure 1 shows a typical chromatogram of clover honey from Egypt, indicating with numbers some selected key volatile compounds. In supplementary material (Figures S1–S6), typical chromatograms of citrus, chestnut, eucalyptus, fir, pine, and thyme honeys from the investigated regions are given.

Clover honeys showed higher amounts (mg/kg) of 2-methylbutanal, 3-methylbutanal, 3-methyl-1-butanol, and 2-methyl-1-butanol compared to the other honey types. The aforementioned compounds comprise isoleucine- and leucine-derived volatiles and are found in a wide range of foods such as honey, beer, cheese, coffee, chicken, fish, chocolate, olive oil, and tea. These volatile compounds are associated with a fruity and malty flavor [21,22].

Citrus honeys were characterized by the higher amounts (mg/kg) of lilac aldehyde C (isomer III), dill ether, methylanthranilate and herboxide (isomer II). These compounds have been reported previously to dominate the volatile profile of citrus honeys among other honey types [3,4].

Chestnut honeys showed the highest amounts (mg/kg) of benzaldehyde, benzeneacetaldehyde, 1-octene, and furfural. The latter volatile compound may serve as an indicator of heat resistance/treatment of chestnut honeys.

Eucalyptus honeys were characterized by the presence of heptane and *beta*-damascenone, since these compounds were found in higher amounts (mg/kg). The presence of hydrocarbons in honey is a typical phenomenon, whereas *beta*-damascenone is a cyclic carotenoid derivative and possesses a rose odor [22].

Fir honeys had higher amounts (mg/kg) of nonanal, decanal, hexanoic acid ethyl ester, heptanoic acid ethyl ester, octanoic acid ethyl ester, nonanoic acid ethyl ester, decanoic acid ethyl ester, dodecanoic acid ethyl ester, tetradecanoic acid ethyl ester, nonane, 5-methyl-4-nonene, 6-methyl-5-hepten-2-one, geranyl acetone, 1-2-(furanyl)-ethanone, *alpha*-isophorone, 4-ketoisophorone, and 2-hydroxyisophorone. The volatile compounds 6-methyl-5-hepten-2-one and geranyl acetone comprise open-chain carotenoid-derived volatiles, whereas the isophorone related compounds belong to the class of norisoprenoids, resulting from the degradation of terpenoids [22]. Geranyl acetone and 6-methyl-5-hepten-2-one possess a strong floral, green and fruit-like odor. In a previous study, these compounds were characterized as exocrine products (cephalic secretions) of cleptoparasitic bees (Holcopasites) [23].

Pine honeys had higher amounts (mg/kg) of acetic acid, octane, *alpha*-pinene and *beta*-thujone. It is remarkable that *beta*-thujone was identified only in pine honeys, comprising a key volatile marker, even though the *p*-value (*p =* 0.053) was slightly higher than the level of confidence considered in the study. This finding is in agreement with a previous study on the botanical differentiation of citrus, fir, pine, and thyme honeys [8]. *Beta*-thujone has a menthol odor [24].

In the case of thyme honeys, 1-octen-3-ol, thymol methyl ether, carvacrol methyl ether, octyl ether, 4,7,7-trimethylbyciclo (3.3.0)-octan-2-one, and eugenol were found in higher amounts (mg/kg) compared to the other honey types. Ethers give an ether-like odor, whereas 1-octen-3-ol provides a floral and grassy odor. Furthermore, 1-Octen-3-ol is referred to as “mushroom alcohol”, given the fact that is the main flavor component of mushrooms [25]. Eugenol is a phenylpropanoid volatile that has a pleasant, spicy, and clove-like odor [26].

### 3.2. Classification of Clover, Citrus, Chestnut, Eucalyptus, Fir, Pine, and Thyme Honeys According to Botanical Origin and the Honey Code Using Volatile Compounds in Combination with Chemometrics

#### 3.2.1. Part I. Classification of Honeys According to Botanical Origin

##### MANOVA and LDA

The four qualitative criteria of the multivariate hypothesis, namely Pillai’s Trace = 5.573 (*F =* 8.698, df = 540, *p* = 0.000, *η^2^* = 0.929), Wilks’ Lambda = 0.000 (*F* = 22.251, df = 540, *p* = 0.000, *η^2^* = 0.972), Hotelling’s Trace = 1034.279 (*F* = 102.151, df = 540, *p* = 0.000, *η^2^* = 0.994), and Roy’s Largest Root = 789.550 (*F =* 526.367, df = 90, *p* = 0.000, *η^2^* = 0.999) showed that there was a statistically significant effect of the botanical origin of honey on the semi-quantitative data of volatile compounds.

More specifically, 62 of the 94 volatile compounds were found to be significant (*p* < 0.05) for the botanical origin differentiation of honeys. Afterwards, these volatiles were subjected to LDA. The minimum tolerance level of the analysis was set at 0.001. Results showed that 4-terpineol, borneol, *para*-cymene, carvacrol methyl ether, thymoquinone, thymol, and eugenol did not pass the tolerance test. Therefore, these volatile compounds were excluded (SPSS program) a priori from the discriminant analysis. Therefore, 56 volatile compounds were subjected to LDA.

Results showed that six canonical discriminant functions were formed: Wilks’ Lambda = 0.000, X^2^ = 1624.974, df = 336, *p* = 0.000 for the first function; Wilks’ Lambda = 0.000, X^2^ = 1049.108, df = 275, *p =* 0.000 for the second function; Wilks’ Lambda = 0.006, X^2^ = 609.334, df = 216, *p* = 0.000 for the third function; Wilks’ Lambda = 0.032, X^2^ = 406.682, df = 159, *p* = 0.000 for the fourth function; Wilks’ Lambda = 0.122, X^2^ = 249.608, df = 104, *p =* 0.000 for the fifth function; and Wilks’ Lambda = 0.409, X^2^ = 106,005, df = 51, *p* = 0.000 for the sixth function.

The first discriminant function recorded the higher eigenvalue (127.977) and a canonical correlation of 0.996, accounting for 71.5% of total variance. The second discriminant function recorded a much lower eigenvalue (39.902) and a canonical correlation of 0.988, accounting for 22.3% of total variance. The third discriminant function recorded a much lower eigenvalue (4.530) and a canonical correlation of 0.905, accounting for 2.5% of total variance. Similarly, the fourth discriminant function recorded an even lower eigenvalue (2.764) and a canonical correlation of 0.857, accounting for 1.5% of total variance. Moreover, the fifth discriminant function had a lower eigenvalue (2.360) and a canonical correlation of 0.838, accounting for 1.3% of total variance. Finally, the sixth discriminant function had the lowest eigenvalue (1.446) and a canonical correlation of 0.769, accounting for 0.8% of total variance. All six discriminant functions accounted for 100% of total variance.

**Figure 2:**
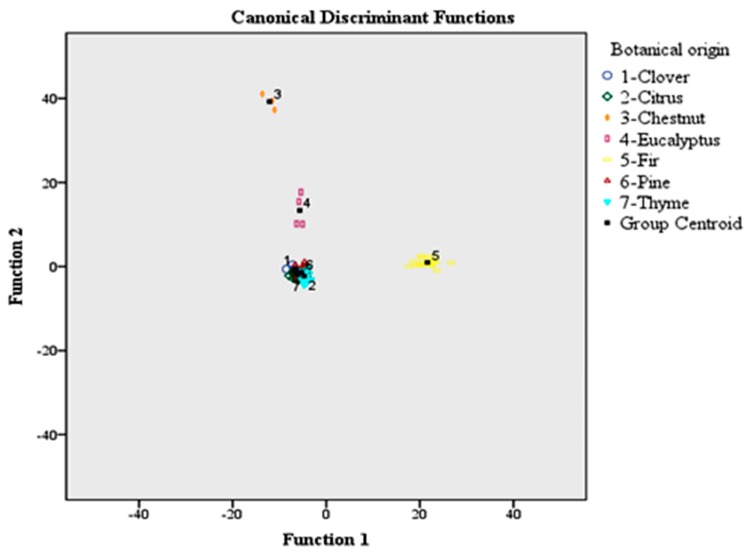
Classification of the 151 honey samples according to botanical origin based on the 56 volatile compounds and LDA. LDA: linear discriminant analysis.

Figure 2 shows the differentiation of honeys according to botanical origin based on the use of 56 volatile compounds and LDA. The group centroid values which represent the unstandardized canonical discriminant functions evaluated at group means are also plotted. Each centroid gives information about the coordinates (discriminant functions) of the group means in the polyparametric space. The abscissa is the first discriminant function, and the ordinate is the second. So, the respective group centroid values were as follows: (−6.609, −0.891), (−6.081, −1.951), (−12.106, 39.255), (−5.664, 13.355), (21.632, 0.974), (−5.265, −1.395), and (−4.712, −2.268) for clover, citrus, chestnut, eucalyptus, fir, pine, and thyme honeys.

The classification rate was 95.4% using the original and 81.5% using the cross-validation method. Supplementary Table S1 shows the significant variables (markers of botanical origin of clover, citrus, chestnut, eucalyptus, fir, pine, and thyme honeys) that were ordered by absolute size of correlation within function. The higher the absolute value of correlation, the best discrimination the variable provides within the discriminant function. The most pronounced markers of the botanical origin of honey are marked with an asterisk. In particular, octanoic acid ethyl ester, nonanoic acid ethyl ester, decanoic acid ethyl ester, dodecanoic acid ethyl ester, decanal, nonanal, 5-methyl-4-nonene, hexanoic acid ethyl ester, heptanoic acid ethyl ester, 4-ketoisophorone, octanol, tetradecanoic acid ethyl ester, geranyl acetone, 6-methyl-5-hepten-2-one, 1-(2-furanyl)-ethanone, *alpha*-isophorone, and decanol contributed to discriminant function 1, whereas hexadecanoic acid ethyl ester contributed to discriminant function 2. It should be remembered that the first two discriminant functions explained 93.8% of total variance.

The botanical origin classification rate of honeys, based on the original method, followed the sequence: clover (87.5%), citrus (90.3%), chestnut (100%), eucalyptus (100%), fir (100%), pine (100%), and thyme (91.4%). In total, 12.5% of clover honeys (one sample) were allocated to pine honeys. In total, 9.7% of citrus honeys (three samples) were allocated to pine honeys. Finally, 8.6% of thyme honeys (three samples) were allocated to pine honeys.

On the contrary, the botanical origin classification rate of honeys, based on the cross-validation method followed the sequence: clover (62.5%), citrus (80.6%), chestnut (66.7%), eucalyptus (75%), fir (100%), pine (94.9%), and thyme (57.1%). In total, 25% of clover honeys (two samples) were allocated to pine honeys, whereas 12.5% of samples (one sample) were allocated to thyme honeys. In total, 19.4% of citrus honeys (six samples) were allocated to pine honeys. In total, 33.3% of chestnut honeys (one sample) were allocated to fir honeys. In total, 25% of eucalyptus honeys (one sample) were allocated to chestnut honeys. In total, 5.2% of pine honeys (two samples) were allocated in equal proportions (2.6%) to clover and chestnut honeys, respectively. Finally, 42.8% of thyme honeys were allocated to clover (11.4%) (four samples), citrus (2.9%) (one sample), chestnut (11.4%) (four samples), and pine (17.1%) (six samples) honeys, respectively (Table 2).

It should be stressed that these results were accepted, given the fact that cross validation is a more pessimistic method of classification of a group of objects, since in cross validation, each case is classified by the functions derived from all cases rather than that particular case. The errors obtained in both classification techniques (original and cross-validation) reveal important findings regarding honey authentication. These errors show the contribution of numerous plants in the produced honeys, even in cases of honeydew honeys. Honeydew honeys are harvested after nectar honeys. Therefore, the contribution of flowering plants in honeydew honeys is quite common.

In addition, present findings may be related to beekeeper practices during the harvesting of different honey types. However, the classification results obtained support previous studies in the literature that focus on honey authentication using volatile compound analysis and chemometrics [3,4,5,8,11]. The results obtained in the present study, which is collective in nature, show a clear differentiation of honeydew vs. nectar honeys.

##### *K*-Nearest Neighbors

For the *k*-NN analysis, the number of samples was randomly assigned to training and holdout partitions. The training sample consisted of 110 honey samples (72.8%), while the holdout sample consisted of 41 samples (27.2%). All cases (151 honey samples) (100%) were used in the statistical analysis, comprising a valid procedure.

The overall classification rate was 77.3% for the training and 87.8% for the holdout sample and was satisfactory in both cases. The botanical origin classification rate of honey types for the training sample followed the sequence: clover (75%), citrus (70.8%), chestnut (0%), eucalyptus (0%), fir (95.8%), pine (92.3%), and thyme (58.3%). However, the zero classification rates of chestnut and eucalyptus honeys are attributed to the limited honey samples, since the majority of them were assigned to the holdout sample. Of the eight clover honey samples subjected to training analysis, six were assigned to clover and two to thyme honeys. Similarly, of the 24 citrus honey samples, 17 samples were assigned to citrus, one to clover, four to pine, and two to thyme honeys, respectively. One chestnut honey sample was assigned to eucalyptus honeys. Of the 24 fir honey samples, 23 were assigned to fir and only one to pine honeys. A similar trend was obtained for pine honeys—in which, 25 samples were assigned to pine and only one to fir honeys, respectively. Finally, of the 24 thyme honey samples, 14 were assigned to thyme, six to pine, three to clover, and one to citrus honeys, respectively.

Regarding the holdout sample classification rate, this was higher than the training sample by 10.5%. The botanical origin classification rate of honey types for the holdout sample followed the sequence: citrus (85.7%), chestnut (0%), eucalyptus (100%), fir (100%), pine (100%), and thyme (81.8%). The clover honeys were assigned previously to training sample. Therefore, no classification results were obtained. Of the seven citrus honey samples subjected to holdout analysis, six were assigned to citrus and one to pine honeys. Of the two chestnut honeys, one was assigned to citrus and one to eucalyptus honeys, respectively. Finally, of the 11 thyme honey samples, nine samples were assigned to thyme and two to pine, respectively (Table 3).

Among the 55 significant volatile compounds (predictors), the most effective predictors (*k*-nearest neighbors) that built the model were acetic acid ethyl ester, formic acid ethyl ester, and 2-methylbutanal. Based on this observation, the *k*-NN analysis was run again by performing feature selection in order to investigate whether the classification results could be improved. The selected features were the aforementioned volatile compounds.

For the specified *k*-nearest neighbors analysis, the sample was divided again to training and holdout partitions. The training sample consisted of 100 honey samples (66.2%), whereas the holdout sample consisted of 51 (33.8%). Both partitions explained 100% of the procedure, indicating that all cases were valid. The analysis stopped when the absolute ratio was less than or equal to the minimum change, which was inserted by default equal to 0.01. The overall classification rate was 83% for the training and 74.5% for the holdout sample. The individual botanical classification rate was differentiated given the fact that the sample assignment to training and holdout partitions was also differentiated (Table 4).

Considering the total standard error of the forced predictors, but also that of the individual predictors, the model built with *k* = 3 (nearest neighbors) was more applicable. The 10 predictors were the three specified predictors, followed by furfural, lilac aldehyde C, benzaldehyde, nonanol, *para*-cymene, 5-methyl-4-nonene, and nonane. The total error rate of the three specified features was 0.74. The respective individual error rate for the seven predictors left was: 0.39, 0.27, 0.25, 0.21, 0.18, 0.17, and 0.17.

**Figure 3:**
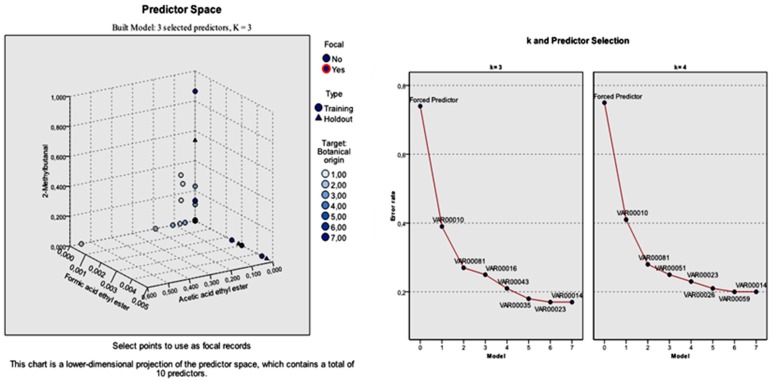
Predictor selection during *k*-NN analysis with *k* = 3 and *k* = 4 nearest neighbors. 1: Clover honeys. 2: Citrus honeys. 3: Chestnut honeys. 4: Eucalyptus honeys. 5: Fir honeys. 6: Pine honeys. 7: Thyme honeys. Forced predictor: Acetic acid ethyl ester, formic acid ethyl ester, 2-methylbutanal. VAR00010: Furfural. VAR00081: Lilac aldehyde C. VAR00016: Benzaldehyde. VAR00043: Nonanol. VAR00035: *para*-Cymene. VAR00023: 5-methyl-4-Nonene. VAR00014: Nonane (Model with *k* = 3). VAR00010: Furfural. VAR00081: Lilac aldehyde C. VAR00051: Phenylethylalcohol. VAR00023: 5-methyl-4-Nonene. VAR00026: 1-Octen-3-ol. VAR00059: Decanol.VAR00014: Nonane (Model with *k* = 4).

On the contrary, the total error rate of the model built with *k* = 4 nearest neighbors in relation to the three specified features was 0.75. The individual error rate for the seven predictors left was: 0.41, 0.28, 0.25, 0.23, 0.21, 0.20, and 0.20 for furfural, lilac aldehyde C, phenylethylalcohol, 5-methyl-4-nonene, 1-octen-3-ol, and nonane, respectively. The selection of the predictors during *k*-NN analysis with *k* = 3 and *k* = 4 nearest neighbors according to the botanical origin of honey is shown in Figure 3.

##### FA

During factor analysis, the 56 significant volatile compounds were reduced to 16 principal components (PCs) based on the rule of an eigenvalue greater than one. The rotated component matrix is given in Supplementary Table S2. The total variance explained was 80.524% (ca. 80.52%).

The fitness of data for factor analysis was estimated by the Kaiser–Meyer–Olkin (KMO) test, which comprises a measure of the effective performance of factor analysis, to a set of data, indicating the sampling adequacy. The acceptable value was considered that of KMO ≥ 0.50. The suitability and applicability of factor analysis was further evaluated using Bartlett’s test of sphericity. This test highlights the hypothesis that the correlation matrix is an identity matrix, which would indicate that the variables incorporated into the model are unrelated and therefore unsuitable for structure detection. Small probability values (*p* < 0.05) indicate that a factor analysis may be useful with data treatment [20]. The value of the KMO test was 0.636. Furthermore, Bartlett’s test of sphericity had the following qualitative values: X^2^ = 10,587.564, df = 1540, and *p* = 0.000.

The factors that best explained the rotated component matrix were the following volatile compounds: Decanol (PC1, 10.454% of total variance), undecanoic acid ethyl ester (PC2, 10.380% of total variance), *para*-cymene (PC3, 7.768% of total variance), 2-hydroxyisophorone (PC4, 7.699% of total variance), nonane (PC5, 5.509% of total variance), dill ether (PC6, 5.058% of total variance), lilac aldehyde C (PC7, 4.412% of total variance), lilac aldehyde D (PC8, 4.352% of total variance), acetic acid ethyl ester (PC9, 3.918% of total variance), 5-methyl-2-phenylhexenal (PC10, 3.905% of total variance), decane (PC11, 3.596% of total variance), 1-(2-furanyl)-ethanone (PC12, 3.563% of total variance), benzeneacetonitrile (PC13, 3.240% of total variance), nonanol (PC14, 2.400% of total variance), 2-methylbutanal (PC15, 2.156% of total variance), and hexanoic acid ethyl ester (PC16, 2.113% of total variance).

#### 3.2.2. Part II. Classification of Honeys According to the Honey Code (CCC-E-F-P-T)

##### MANOVA and LDA

As in the case of the botanical origin differentiation of honeys, the four qualitative criteria of the multivariate hypothesis had the following values: Pillai’s Trace = 3.770 (*F* = 10.640, df = 364, *p* = 0.000, *η^2^* = 0.943), Wilks’ Lambda = 0.000 (*F* = 23.673, df = 364, *p* = 0.000, *η^2^* = 0.974), Hotelling’s Trace = 431.692 (*F = 64.635*, df = 364, *p* = 0.000, *η2* = 0.991), and Roy’s Largest Root = 361.350 (*F* = 234.282, df = 91, *p* = 0.000, *η^2^* = 0.997) showed that there was a statistically significant effect of the honey code on the semi-quantitative data of volatile compounds. More specifically, 65 of the 94 volatile compounds were found to be significant (*p* < 0.05) for the classification of honeys according to the honey code. Then, these volatiles were subjected to LDA. Results showed that 4-terpineol, (1R)-1,7,7-trimethyl-bicyclo-(2.2.1)-heptan-2-one, carvacrol methyl ether, thymoquinone, thymol, and eugenol did not pass the tolerance test. Therefore, these volatile compounds were excluded from the discriminant analysis. In that sense, 59 volatile compounds contributed to the structure matrix as shown in Supplementary Table S3.

Results showed that four canonical discriminant functions were formed: Wilks’ Lambda = 0.000, X^2^ = 1009.601, df = 236, *p* = 0.000 for the first function; Wilks’ Lambda = 0.014, X^2^ = 502.352, df = 174, *p = 0.000* for the second function; Wilks’ Lambda = 0.090, X^2^ = 284.776, df = 114, *p = 0.000* for the third function; and Wilks’ Lambda = 0.369, X^2^ = 117.597, df = 56, *p* = 0.000 for the fourth function. The first discriminant function recorded the higher eigenvalue (72.605) and a canonical correlation of 0.993, accounting for 87.7% of total variance. The second discriminant function recorded a much lower eigenvalue (5.321) and a canonical correlation of 0.917, accounting for 6.4% of total variance. The third discriminant function recorded a lower eigenvalue (3.124) and a canonical correlation of 0.87o, accounting for 3.8% of total variance. Similarly, the fourth discriminant function recorded the lowest eigenvalue (1.709) and a canonical correlation of 0.794, accounting for 2.1% of total variance. All four discriminant functions accounted for 100% of total variance.

**Figure 4:**
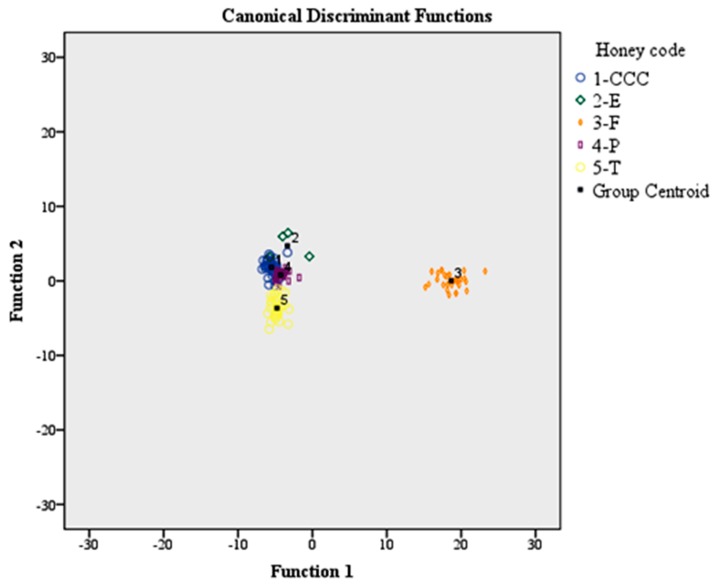
Classification of the 151 honey samples according to the honey code based on 57 volatile compounds and LDA.

In Figure 4, the differentiation of the 151 honey samples according to the honey code is shown. The group centroid values are as follows: (−4.968, −1.828), (−2.691, −4.647), (16.454, −0.115), (−4.012, −0.914), (−3.834, 3.844) for honeys encoded with CCC, E, F, P, and T, respectively.

The classification rate was 96.0% using the original and 86.1% using the cross-validation method. The classification rate of honeys according to the honey code, based on the original method, followed the sequence: CCC (88.1%), E (100%), F (100%), P (100%), and T (97.1%). In total, 12.9% of CCC honeys were allocated to the E group (4.8%, two samples) and to the P group (7.1%, three samples). In total, 2.9% (three samples) of T honeys were allocated to the P group.

For the cross-validation method, the classification rate of honeys followed the sequence: CCC (81%), E (75%), F (93.5%), P (92.3%), and T (80%). In total, 4.8% (two samples) of CCC honeys were allocated to the E group; 2.4% (one sample) were allocated to the F group, and 11.9% of samples (five samples) were allocated to the P group. In total, 25% (one sample) of E honeys were allocated to CCC honeys. In total, 3.2% of F honeys were allocated in equal proportions to the E (one sample) and P (one sample) groups, respectively. The same holds for P honeys—in which, two samples (5.2% of the total population) were allocated to the E (one sample, 2,6%) and F (one sample, 2,6%) groups, respectively. Finally, 14.3% of T honeys were allocated to P honeys (five samples), and 5.7% (two samples) to E honeys (Table 5).

As can be observed, the classification of honeys according to the honey code based on original and cross validation methods provided higher prediction rates compared to the differentiation of honeys according to botanical origin. In Supplementary Table S3, the key volatile compounds for the discrimination of honeys according to the honey code are marked with an asterisk.

##### *K*-Nearest Neighbors

The training sample consisted of 111 honey samples that represented 73.5% of the total sample population. Similarly, the holdout sample consisted of 40 samples that represented 26.5% of the sample population. All cases (151 honey samples) (100%) were used in the statistical analysis, comprising a valid procedure. The scale features (predictors) (57 statistically significant volatile compounds) were normalized during analysis.

The overall classification rate was 81.1% for the training and 80% for the holdout sample and was satisfactory in both cases. The classification rate of honey types according to the honey code followed the sequence: CCC (86.7%), E (25%), F (96.2%), P (96.4%), and T (47.8%). Of the 30 CCC samples subjected to training analysis, one sample was assigned to the E group and three samples to the P group. Similarly, of the four E honey samples, three honey samples were assigned to the CCC group and only one sample to the E group. Of the 26 F honeys, 25 samples were assigned to the F group and only one sample to the P group. Of the 28 P honeys, 27 samples were assigned to the P group and only one sample to the F group. Finally, of the 23 T honey samples, 11 were assigned to T, six to CCC, and six to P, respectively. For the training sample, the honey code had the following hierarchy: CCC > E, F, P > T, CCC > T, and was in general applicable.

The classification rates for the holdout sample were improved for the F, P, and T honey groups. The classification rate of honey samples according to the honey code for the holdout sample followed the sequence: CCC (83.3%), F (100%), P (100%), and T (50%). As can be observed, the hierarchy in honey lettering was maintained by 3/5 cases (F, P > T and CCC > T). Of the 12 CCC honey samples assigned to the holdout sample, 10 were assigned to the CCC group and two to the P group. F and P honey samples were perfectly assigned to the respective groups. Finally, of the 12 T honey samples, seven were assigned to T group, two to the CCC, and four to the P group (Table 6).

Among the 57 significant volatile compounds (predictors), the most effective predictors (*k*-nearest neighbors) that built the model were formic acid ethyl ester, acetic acid ethyl ester, and heptane. Therefore, the *k*-NN analysis was repeated by performing feature selection in order to investigate whether the classification results could be improved. The selected features were the aforementioned volatile compounds.

For the specified *k*-nearest neighbors, the sample was divided again to the training and holdout partitions. The training sample consisted of 110 honey samples (72.8%), whereas the rest of the honey samples represented the holdout sample (27.2%). Similar to the botanical origin differentiation of honeys, the analysis stopped when the absolute ratio was less than or equal to the minimum change which was inserted by default equal to 0.01.

The overall classification rate was 89.1% for the training and 63.4% for the holdout sample. The classification rate of the training sample was improved, whereas that of the holdout sample was decreased. However, discrepancies among the two methods followed (simple *k*-NN and *k*-NN analysis with feature selection) may be attributed to the number of the predictors assigned to the model and the random dividing of the sample to training and holdout partitions in relation to sample size.

The classification rate of honey samples according to the honey code for the training sample followed the sequence: CCC (87.5%), E (0%), F (100%), P (93.1%), and T (88.5%). These results show that the honey code was satisfactorily applicable given the hierarchy followed: CCC > E, F > P > T and CCC > T. The classification rate of honey samples according to the honey code for the holdout sample followed the sequence: CCC (50%), E (0%), F (72.7%), P (80%), and T (55.6%). Similarly, the honey code was satisfactorily applicable given the hierarchy followed: CCC > E, F, P > T, and CCC > T. The classification results along with each sample assignment are given in Table 7.

The model was built with *k* = 3, *k* = 4, and *k* = 5 nearest neighbors, which were automatically selected. Similar to the botanical origin differentiation of honeys, the forced predictors’ error was considered for the selection of the best model. The 10 predictors that were obtained during the *k*-NN analysis with *k* = 3 neighbors were the three forced predictors (heptane, formic acid ethyl ester, acetic acid ethyl ester) followed by dodecanoic acid ethyl ester, benzaldehyde, nonanal, *alpha*-terpinene, geranyl acetone, 5-methyl-4-nonene, and lilac aldehyde B (isomer II). The total error rate of the three specified features was 0.6818. The respective individual error rate for the seven aforementioned predictors was: 0.30, 0.2727, 0.2455, 0.1818, 0.1616, 0.1455, 0.1364, and 0.1364.

On the other hand, the total error rate of the model built with *k* = 4 nearest neighbors in relation to the three specified features was 0.6636. The individual error rate for the six predictors left was: 0.3000, 0.1909, 0.1636, 0.1364, 0.1273, and 0.1273, for furfural, lilac aldehyde C (isomer III), decanoic acid, lilac aldehyde D (isomer IV), pentanoic acid, and nonane.

**Figure 5:**
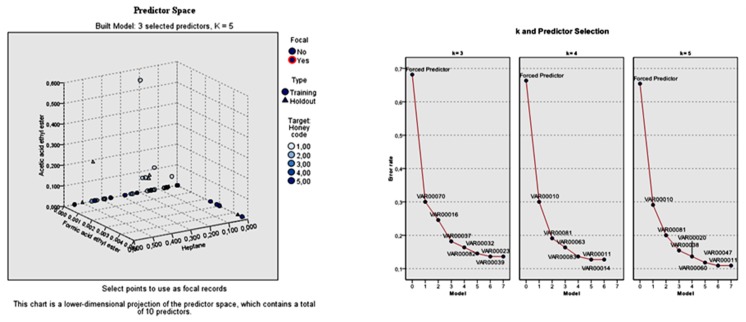
Predictor selection during *k*-NN analysis with *k* = 3, *k* = 4, and *k* = 5 nearest neighbors. 1: CCC. 2: E. 3: F. 4: P. 5: T. Forced predictor: Heptane, formic acid ethyl ester, acetic acid ethyl ester. VAR00070: Dodecanoic acid ethyl ester. VAR00016: Benzaldehyde. VAR00037: Nonanal. VAR00032: *Alpha*-terpinene. VAR00082: Geranyl acetone. VAR00023: 5-Methyl-4-Nonene. VAR00039: Lilac aldehyde B (isomer II). (Model with *k* = 3); VAR00010: Furfural. VAR00081: Lilac aldehyde C (isomer III). VAR00063: Decanoic acid. VAR00083: Lilac aldehyde D (isomer IV). VAR00011: Pentanoic acid. VAR00014: Nonane (Model with *k* = 4); VAR00010: Furfural. VAR00081: Lilac aldehyde C (isomer III). VAR00020: Benzeneacetaldehyde. VAR00038: Octanoic acid ethyl ester. VAR00060: Nonanoic acid ethyl ester. VAR00047: Nonanoic acid. VAR00011: Pentanoic acid (Model with *k* = 5).

Finally, the total error rate of the model built with *k* = 5 nearest neighbors in relation to the three specified features was 0.6545. The individual error rate for the seven predictors left was: 0.2909, 0.20, 0.1364, 0.1545, 0.1182, 0.1091, and 0.1091, for furfural, lilac aldehyde C (isomer III), benzeneacetaldehyde, octanoic acid ethyl ester, nonanoic acid ethyl ester, nonanoic acid, and pentanoic acid. The selection of the predictors during *k*-NN analysis with *k* = 3, *k* = 4, and *k* = 5 nearest neighbors according to the honey code is shown in Figure 5.

##### FA

The 59 significant volatile compounds were reduced to 15 principal components (PCs) based on the rule of an eigenvalue greater than one. The rotated component matrix is given in Supplementary Table S4. The total variance explained was 81.547% (ca. 81.55%). As can be observed, the total variance explained of samples according to the honey code was higher than those that were grouped according to botanical origin. In addition, the KMO test had a higher value: KMO = 0.615. Furthermore, Bartlett’s test of sphericity had the following qualitative values: X^2^ = 12,376.576, df = 1596, *p =* 0.000. The factors that best explained the rotated component matrix were the following volatile compounds: *Para*-cymene (PC1, 12.207% of total variance), undecanoic acid ethyl ester (PC2, 10.520% of total variance), decanol (PC3, 10.03% of total variance), 2-hydroxyisophorone (PC4, 7.788% of total variance), decanoic acid (PC5, 7.386% of total variance), dill ether (PC6, 5.078% of total variance), 1-(2-furanyl)-ethanone (PC7, 4.506% of total variance), undecane (PC8, 4.326% of total variance), lilac aldehyde B (PC9, 3.254% of total variance), dodecanoic acid (PC10, 2.989% of total variance), lilac aldehyde D (PC11, 2.986% of total variance), benzeneacetonitrile (PC12, 2.904% of total variance), decanoic acid ethyl ester (PC13, 2.821% of total variance), 4,7,7-trimethylbyciclo (3.3.0)-octan-2-one (PC14, 2.819% of total variance), and dodecanoic acid ethyl ester (PC15, 1.960% of total variance).

## 4. Conclusions

Results of the present study showed that specific volatile compounds in combination with polyparametric statistical techniques such as MANOVA, LDA, *k*-NN and FA, may provide exhaustive information about the botanical origin of honey. Even though honey samples were harvested in different parts of the world, the classification of honeys according to botanical origin was, in general, very satisfactory. At the same time, the application of the honey code to the collective set of data and the use of the aforementioned statistical techniques resulted in a higher classification rate of the honey samples. The use of hierarchical classification strategies (HCSs) may expand the state of the art and flourish the complicated topic of “Honey authentication” by highlighting with numbers the distinction/differentiation rates, for instance, of monofloral or blends of nectar or honeydew honeys with specific and intense flavors.

## Figures and Tables

**Table 1:** Semi-quantitative results of volatile compounds tentatively identified in honey samples according to botanical origin.

RT ^a^ (min)	Volatile Compounds (mg/kg)	RIexp	Clover	Citrus	Chestnut	Eucalyptus	Fir	Pine	Thyme	*F*	*p*
**Acids**											
5.40	Formic acid	<800	ni	0.02 (0.09)	ni	ni	ni	0.01 (0.04)	0.02 (0.09)	0.312	0.930
6.53	Acetic acid	<800	ni	0.04 (0.16)	ni	ni	0.07 (0.22)	0.16 (0.65)	0.05 (0.17)	0.570	0.753
13.02	Pentanoic acid	823	ni	ni	ni	ni	ni	0.0004	0.002	3.034	0.008
16.36	Hexanoic acid	956	ni	ni	ni	ni	ni	0.01 (0.06)	ni	0.572	0.752
18.57	Heptanoic acid	1053	ni	ni	ni	ni	ni	0.0001 (0.0005)	ni	0.468	0.831
20.65	Octanoic acid	1151	ni	ni	ni	ni	ni	0.03 (0.13)	ni	1.097	0.367
22.60	Nonanoic acid	1249	ni	ni	ni	ni	ni	0.03 (0.11)	ni	1.722	0.120
24.42	Decanoic acid	1348	ni	ni	ni	ni	ni	0.01 (0.03)	ni	1.624	0.144
27.79	Dodecanoic acid	1549	ni	ni	ni	ni	ni	0.002 (0.004)	ni	3.488	0.003
**Aldehydes**											
6.80	2-methyl-Butanal	<800	0.09 (0.14)	0.02 (0.05)	ni	ni	ni	ni	0.04 (0.17)	1.876	0.089
8.35	3-methyl-Butanal	<800	0.06 (0.05)	0.02 (0.01)	ni	ni	ni	ni	0.01 (0.05)	6.548	0.000
13.36	Furfural	836	0.12 (0.11)	0.06 (0.14)	0.95 (1.57)	ni	0.62 (0.31)	0.12 (0.51)	0.01 (0.02)	12.404	0.000
16.63	5-methyl-2-Furaldehyde	967	ni	0.002 (0.011)	ni	ni	ni	0.013 (0.057)	0.005 (0.02)	0.685	0.662
16.86	Benzaldehyde	977	ni	0.02 (0.03)	0.27 (0.23)	0.07 (0.08)	0.22 (0.31)	0.05 (0.18)	0.07 (0.07)	4.966	0.000
17.51	Octanal	1005	0.05 (0.09)	0.003 (0.01)	0.26 (0.34)	0.03 (0.03)	0.36 (0.34)	0.08 (0.40)	0.01 (0.02)	6.607	0.000
18.66	Benzeneacetaldehyde	1059	ni	0.06 (0.10)	0.38 (0.33)	0.05 (0.05)	0.31 (0.40)	0.05 (0.14)	0.31 (0.44)	5.009	0.000
19.75	Nonanal	1107	0.09 (0.16)	0.08 (0.09)	1.29 (2.01)	ni	3.30 (3.28)	0.17 (0.35)	0.03 (0.05)	18.021	0.000
20.58	Lilac aldehyde A (isomer I)	1145	ni	0.09 (0.16)	ni	ni	ni	ni	0.01 (0.03)	6.584	0.000
20.60	Lilac aldehyde B (isomer II)	1154	ni	0.11 (0.17)	ni	0.01 (0.02)	ni	ni	0.003 (0.01)	8.820	0.000
20.78	Lilac aldehyde C (isomer III)	1172	ni	0.34 (0.32)	0.20 (0.34)	ni	ni	ni	0.02 (0.03)	19.726	0.000
21.23	Lilac aldehyde D (isomer IV)	1178	ni	0.01 (0.03)	0.10 (0.17)	ni	ni	ni	ni	9.517	0.000
21.83	Decanal	1209	ni	0.02 (0.05)	0.89 (1.44)	0.03 (0.02)	1.43 (1.28)	ni	0.01 (0.02)	22.745	0.000
22.86	2-methyl-3-phenylPropanal	1245	ni	ni	ni	ni	ni	ni	0.004 (0.03)	0.542	0.775
23.06	4-methoxy-Benzaldehyde	1252	ni	ni	ni	ni	ni	ni	0.02 (0.01)	0.542	0.775
27.11	5-methyl-2-phenyl-2-Hexenal	1475	ni	ni	ni	ni	ni	ni	0.004 (0.001)	2.936	0.010
**Alcohols**											
10.45	3-methyl-1-Butanol	<800	0.034 (0.05)	0.006 (0.034)	ni	ni	ni	ni	0.01 (0.06)	1.436	0.205
10.56	2-methyl-1-Butanol	<800	0.02 (0.04)	ni	ni	ni	ni	ni	0.001 (0.003)	6.671	0.000
16.80	1-Octen-3-ol	979	ni	ni		ni	ni	ni	0.42 (1.17)	2.396	0.031
17.20	3-Octanol	992	ni	ni	ni	ni	ni	ni	0.01 (0.06)	0.542	0.775
17.91	2-ethyl-1-Hexanol	1027	ni	0.001 (0.005)	0.04 (0.04)	0.13 (0.06)	0.30 (0.32)	0.05 (0.16)	0.001 (0.002)	11.588	0.000
18.94	1-Octanol	1069	ni	ni	0.10 (0.18)	ni	0.15 (0.22)	0.02 (0.08)	ni	7.271	0.000
20.15	Phenylethylalcohol	1129	ni	ni	ni	ni	ni	ni	0.05 (0.10)	4.820	0.000
21.07	1-Nonanol	1171	ni	ni	0.47 (0.55)	0.04 (0.05)	ni	0.14 (0.45)	ni	3.271	0.005
23.02	1-Decanol	1271	ni	ni	ni	ni	0.08 (0.24)	0.001 (0.004)	ni	2.258	0.041
**Esters**											
5.19	Formic acid ethyl ester	<800	ni	ni	ni	ni	ni	ni	0.006 (0.001)	3.559	0.003
7.18	Acetic acid ethyl ester	<800	0.13 (0.18)	0.01 (0.02)	ni	ni	ni	ni	ni	11.814	0.000
17.16	Hexanoic acid ethyl ester	996	ni	ni	0.09 (0.10)	0.08 (0.03)	0.28 (0.34)	0.01 (0.01)	0.02 (0.04)	11.742	0.000
19.47	Heptanoic acid ethyl ester	1097	ni	ni	0.04 (0.03)	0.01 (0.02)	0.21 (0.31)	0.07 (0.01)	ni	8.949	0.000
21.38	Octanoic acid ethyl ester	1193	ni	0.01 (0.02)	0.46 (0.37)	0.22 (0.16)	1.08 (0.42)	0.10 (0.21)	0.07 (0.10)	80.095	0.000
23.27	Nonanoic acid ethyl ester	1298	ni	0.03 (0.04)	0.69 (0.71)	0.28 (0.10)	2.11 (1.14)	0.19 (0.44)	0.05 (0.07)	49.997	0.000
24.74	Methyl anthranilate	1366	ni	0.02 (0.04)	ni	ni	ni	ni	ni	5.918	0.000
25.05	Decanoic acid ethyl ester	1389	0.04 (0.06)	0.02 (0.05)	0.48 (0.63)	0.05 (0.06)	0.99 (0.55)	0.07 (0.15)	0.03 (0.06)	49.415	0.000
26.87	Undecanoic acid ethyl ester	1491	ni	ni	0.02 (0.03)	ni	0.02 (0.03)	ni	ni	7.569	0.000
28.46	Dodecanoic acid ethyl ester	1591	ni	ni	0.22 (0.28)	0.05 (0.01)	0.59 (0.38)	0.03 (0.07)	ni	29.067	0.000
29.96	Tridecanoic acid ethyl ester	1791	ni	ni	0.23 (0.40)	ni	0.04 (0.19)	0.02 (0.10)	ni	2.358	0.033
31.31	Tetradecanoic acid ethyl ester	1883	ni	ni	0.14 (0.24)	0.004 (0.01)	0.22 (0.40)	ni	ni	5.991	0.000
34.78	Hexadecanoic acid ethyl ester	1982	ni	ni	0.02 (0.02)	0.01 (0.01)	ni	ni	ni	35.197	0.000
**Ethers**											
21.72	Dill ether	1203	ni	0.07 (0.07)	ni	ni	ni	ni	0.01 (0.02)	18.039	0.000
22.27	Thymol methyl ether [Benzene, 2-methoxy-4-methyl-1-(1-methylethyl)-]	1235	ni	ni	ni	ni	ni	ni	0.10 (0.27)	2.534	0.023
22.48	Carvacrol methyl ether	1246	ni	ni	ni	ni	ni	ni	0.05 (0.17)	1.797	0.104
29.52	Octyl ether	1617	ni	ni	ni	ni	ni	ni	0.02 (0.08)	1.374	0.229
**Hydrocarbons**											
9.49	Heptane	<800	0.24 (0.11)	0.04 (0.05)	0.26 (0.22)	0.41 (0.11)	ni	ni	0.11 (0.18)	21.131	0.000
12.18	1-Octene	<800	ni	ni	0.36 (0.31)	ni	0.30 (0.36)	0.15 (0.89)	ni	1.616	0.147
12.27	Octane	800	ni	0.01 (0.02)	0.66 (0.32)	0.96 (0.59)	ni	1.35 (8.01)	0.04 (0.08)	0.512	0.798
14.81	1-Nonene	889	ni	ni	ni	ni	ni	0.18 (0.98)	ni	0.615	0.718
15.02	Nonane	900	ni	ni	0.20 (0.27)	0.05 (0.04)	0.53 (0.36)	0.18 (0.98)	ni	3.681	0.002
16.98	5-methyl-4-Nonene	981	ni	ni	0.06 (0.11)	ni	0.25 (0.29)	0.001 (0.003)	ni (0.001)	13.789	0.000
17.39	Decane	1000	ni	ni	0.03 (0.02)	0.03 (0.01)	ni	0.002 (0.003)	ni	76.961	0.000
19.61	Undecane	1100	ni	ni	ni	ni	ni	ni	0.001 (0.003)	2.132	0.053
**Ketones**											
15.37	1-(2-furanyl)-Ethanone	914	ni	ni	0.08 (0.14)	ni	0.14 (0.32)	ni	0.01 (0.05)	3.546	0.003
16.76	6-methyl-5-Hepten-2-one	973	ni	ni	0.01 (0.01)	0.01 (0.01)	0.02 (0.04)	ni	ni	4.139	0.001
17.97	(*1S,4S,5R*)-4-methyl-1-propan-2-ylbicyclo(3.1.0)-hexan-3-one (beta-Thujone)	1125	ni	ni	ni	ni	ni	0.04 (0.01)	ni	2.134	0.053
21.10	(*1R,4S*)-1,7,7-trimethylbyciclo-(2.2.1)-heptan-2-one (Camphor)	1143	ni	ni	ni	ni	ni	ni	0.08 (0.21)	2.425	0.029
24.83	3-hydroxy-4-phenyl-Butanone	1347	ni	ni	ni	ni	ni	ni	0.01 (0.03)	1.851	0.093
25.37	1-(2,6,6-trimethyl-1,3-cyclohexadien-1-yl)-2-Buten-1-one *(beta*-Damascenone)	1359	ni	ni	0.01 (0.01)	0.08 (0.04)	0.01 (0.03)	0.03 (0.14)	0.002 (0.003)	1.102	0.364
26.27	(*E*)-6,10-dimethyl-5,9-Undecadien-2-one (Geranyl acetone)	1455	ni	ni	ni	ni	0.01 (0.03)	ni	ni	5.276	0.000
27.96	4,7,7-trimethylbyciclo(3.3.0)-octan-2-one	1659	ni	ni	ni	ni	ni	ni	0.02 (0.01)	1.670	0.132
**Norisoprenoids and quinones**											
20.41	3,5,5-trimethyl-2-Cyclohexen-1-one (*alpha*-Isophorone)	1139	ni	ni	0.01 (0.02)	ni	0.02 (0.07)	ni	ni	2.500	0.025
20.84	2,6,6-trimethyl-Cyclohex-2-ene-1,4-dione (4-Ketoisophorone)	1160	ni	ni	ni	0.03 (0.04)	0.25 (0.38)	ni	ni	8.373	0.000
20.98	2-hydroxy-3,5,5-trimethyl-2-Cyclohex-2-enone (2-hydroxyIsophorone)	1167	ni	ni	0.01 (0.01)	0.08 (0.05)	0.12 (0.20)	ni	ni	7.102	0.000
22.83	2-methyl-5-propan-2-ylcyclohexa-2,5-diene-1,4-dione (Thymoquinone)	1250	ni	ni	ni	ni	ni	ni	0.51 (1.25)	3.178	0.006
**Terpenoids**											
16.18	2,6,6-trimethylbicyclo[3.1.1]Hept-2-ene (*α*- Pinene)	948	ni	ni	0.04 (0.02)	0.04 (0.02)	0.09 (0.24)	0.11 (0.54)	0.06 (0.02)	0.681	0.665
17.57	Herboxide second isomer	1005	ni	0.06 (0.08)	0.02 (0.04)	ni	ni	ni	0.04 (0.02)	10.175	0.000
17.97	1-methyl-4-propan-2-ylCyclohexa-1,3-diene (*α*-Terpinene)	1026	ni	ni	ni	ni	ni	ni	0.03 (0.10)	1.623	0.145
18.26	1-methyl-4-prop-1-en-2-ylCyclohexene (dl-Limonene)	1031	ni	ni	ni	ni	0.02 (0.05)	ni	0.04 (0.10)	1.935	0.079
18.38	4-methylene-1-(1-methylethyl)bicycle(3.1.0)-Hexane (Sabinene)	1044	ni	ni	ni	ni	ni	ni	0.05 (0.17)	1.645	0.139
18.43	1,8-Cineole (Eucalyptol)	1047	ni	ni	ni	ni	ni	ni	0.58 (2.16)	1.386	0.224
18.85	1-methyl-4-propan-2-ylcyclohexa-1,4-diene (γ-Terpinene)	1056	ni	ni	ni	ni	ni	ni	0.29 (0.86)	2.198	0.047
19.14	*cis*-Linalool oxide	1073	ni	0.05 (0.06)	0.17 (0.29)	0.06(0.07)	0.11 (0.13)	ni	0.03 (0.06)	7.502	0.000
19.54	3,7-dimethyl-1,6-Octadien-3-ol (Linalool)	1088	ni	0.05 (0.04)	ni	0.30(0.11)	0.14 (0.25)	ni	0.40 (1.17)	1.789	0.105
19.59	1-methyl-4-propan-2-yl-Cyclohexa-1,3-diene (*α*-Terpinolene)	1090	ni	0.001 (0.005)	0.20 (0.18)	ni	ni	ni	0.03 (0.09)	10.265	0.000
19.63	Hotrienol	1104	ni	0.01 (0.02)	ni	0.10(0.20)	ni	ni	0.06 (0.14)	3.323	0.004
21.54	(*1S*-endo)-1,7,7-trimethyl-bicyclo(2.2.1)-Heptan-2-ol (Borneol)	1169	ni	ni	0.02 (0.03)	ni	ni	ni	0.19 (0.48)	2.860	0.012
21.61	4-methyl-1-propan-2-ylCyclohex-3-en-1-ol (Terpinen-4-ol)	1178	ni	ni	ni	ni	ni	ni	0.39 (0.90)	3.519	0.003
21.84	2-(4-methylcyclohex-3-en-1-yl)Propan-2-ol (*α*-Terpineol)	1191	ni	0.004 (0.02)	ni	ni	ni	ni	0.04 (0.17)	0.838	0.543
22.25	*alpha*,4-dimethyl-cyclohex-3-ene-1-Acetaldehyde (*para*-Menth-1-en-9-al)	1231	ni	0.04 (0.05)	ni	ni	ni	ni	0.04 (0.02)	11.818	0.000
26.32	*1R,4E*,9S)-4,11,11-Trimethyl-8-methylidenebicyclo(7.2.0)-undec-4-ene (Caryophyllene)	1418	ni	ni	ni	ni	ni	ni	0.03 (0.12)	1.243	0.288
**Phenolic and benzene derivatives**											
18.24	1-methyl-4-(1-methylethyl)Benzene *(para*-Cymene)	1038	ni	0.03 (0.04)	ni	0.03 (0.04)	ni	ni	1.84 (5.00)	2.528	0.023
20.64	Benzeneacetonitrile	1150	ni	ni	ni	ni	ni	ni	0.03 (0.07)	2.799	0.013
23.31	5-methyl-2-(1-methylethyl)Phenol (Thymol)	1296	ni	0.006 (0.003)	ni	ni	ni	ni	3.51 (8.63)	3.133	0.006
23.58	3-methyl-4-isopropylPhenol	1308	ni	ni	ni	ni	ni	ni	0.003 (0.01)	1.335	0.245
23.68	2-methyl-5-(1-methylethyl)Phenol (Carvacrol)	1309	ni	0.01 (0.04)	ni	ni	ni	ni	0.001 (0.002)	0.777	0.590
24.11	3,4,5-trimethylPhenol	1331	ni	ni	0.10 (0.17)	ni	0.10 (0.25)	ni	ni	3.609	0.002
24.73	2-methoxy-4-(2-propenyl)Phenol (Eugenol)	1359	ni	ni	ni	ni	ni	ni	0.12 (0.32)	2.428	0.029

^a^ RT: Retention time. RIexp: Experimental retention index values using standard hydrocarbons naturally present in honey. ni: not identified. The non-identified volatile compounds were treated as zeros for chemometrics and not as missing values. *F*: Values of the F distribution. *p*: probability.

**Table 2:** Discriminatory power of the LDA model based on the significant volatile compounds according to the botanical origin of honey.

Chemometric Technique	Prediction Rate	Botanical Origin	Predicted Group Membership							Total Honey Samples
LDA	%		Clover	Citrus	Chestnut	Eucalyptus	Fir	Pine	Thyme	
Original ^a^	Count	Clover	7	0	0	0	0	1	0	8
		Citrus	0	28	0	0	0	3	0	31
		Chestnut	0	0	3	0	0	0	0	3
		Eucalyptus	0	0	0	4	0	0	0	4
		Fir	0	0	0	0	31	0	0	31
		Pine	0	0	0	0	0	39	0	39
		Thyme	0	0	0	0	0	3	32	35
	%	Clover	87.5	0.0	0.0	0.0	0.0	12.5	0.0	100.0
		Citrus	0.0	90.3	0.0	0.0	0.0	9.7	0.0	100.0
		Chestnut	0.0	0.0	100.0	0.0	0.0	0.0	0.0	100.0
		Eucalyptus	0.0	0.0	0.0	100.0	0.0	0.0	0.0	100.0
		Fir	0.0	0.0	0.0	0.0	100.0	0.0	0.0	100.0
		Pine	0.0	0.0	0.0	0.0	0.0	100.0	0.0	100.0
		Thyme	0.0	0.0	0.0	0.0	0.0	8.6	91.4	100.0
Cross validated ^b,c^	Count	Clover	5	0	0	0	0	2	1	8
		Citrus	0	25	0	0	0	6	0	31
		Chestnut	0	0	2	0	1	0	0	3
		Eucalyptus	0	0	1	3	0	0	0	4
		Fir	0	0	0	0	31	0	0	31
		Pine	1	0	0	0	1	37	0	39
		Thyme	4	1	4	0	0	6	20	35
	%	Clover	62.5	0.0	0.0	0.0	0.0	25.0	12.5	100.0
		Citrus	0.0	80.6	0.0	0.0	0.0	19.4	0.0	100.0
		Chestnut	0.0	0.0	66.7	0.0	33.3	0.0	0.0	100.0
		Eucalyptus	0.0	0.0	25.0	75.0	0.0	0.0	0.0	100.0
		Fir	0.0	0.0	0.0	0.0	100.0	0.0	0.0	100.0
		Pine	2.6	0.0	0.0	0.0	2.6	94.9	0.0	100.0
		Thyme	11.4	2.9	11.4	0.0	0.0	17.1	57.1	100.0

^a^ 95.4% of grouped cases using the original method were correctly classified. ^b^ Cross validation was performed only for those cases in the analysis. In cross validation, each case is classified by the functions derived from all cases rather than that particular case. ^c^ 81.5% of cross-validated grouped cases were correctly classified.

**Table 3:** Classification of clover, citrus, chestnut, eucalyptus, fir, pine, and thyme honeys according to botanical origin using the 56 volatile compounds and *K*-Nearest Neighbors (*k*-NN) analysis.

Partition	Observed	Predicted							
		Clover	Citrus	Chestnut	Eucalyptus	Fir	Pine	Thyme	Percent Correct
Training	Clover	6	0	0	0	0	0	2	75.0%
	Citrus	1	17	0	0	0	4	2	70.8%
	Chestnut	0	0	0	1	0	0	0	0.0%
	Eucalyptus	0	0	0	0	0	0	3	0.0%
	Fir	0	0	0	0	23	1	0	95.8%
	Pine	0	0	0	0	1	25	0	96.2%
	Thyme	3	1	0	0	0	6	14	58.3%
	Overall Percent	9.1%	16.4%	0.0%	0.9%	21.8%	32.7%	19.1%	77.3%
Holdout	Clover	0	0	0	0	0	0	0	
	Citrus	0	6	0	0	0	1	0	85.7%
	Chestnut	0	1	0	1	0	0	0	0.0%
	Eucalyptus	0	0	0	1	0	0	0	100.0%
	Fir	0	0	0	0	7	0	0	100.0%
	Pine	0	0	0	0	0	13	0	100.0%
	Thyme	0	0	0	0	0	2	9	81.8%
	Missing	0	0	0	0	0	0	0	
	Overall Percent	0.0%	17.1%	0.0%	4.9%	17.1%	39.0%	22.0%	87.8%

**Table 4:** Classification of clover, citrus, chestnut, eucalyptus, fir, pine, and thyme honeys according to botanical origin using the 10 volatile compounds and *k*-NN.

Partition	Observed	Predicted							
		Clover	Citrus	Chestnut	Eucalyptus	Fir	Pine	Thyme	Percent Correct
Training	Clover	5	1	0	0	0	1	0	71.4%
	Citrus	0	21	0	0	0	0	1	95.5%
	Chestnut	0	0	0	0	1	1	1	0.0%
	Eucalyptus	0	1	0	0	0	1	1	0.0%
	Fir	0	0	0	0	17	0	0	100.0%
	Pine	0	0	0	0	1	25	0	96.2%
	Thyme	1	4	0	0	0	2	15	68.2%
	Overall Percent	6.0%	27.0%	0.0%	0.0%	19.0%	30.0%	18.0%	83.0%
Holdout	Clover	0	1	0	0	0	0	0	0.0%
	Citrus	0	5	0	0	0	4	0	55.6%
	Chestnut	0	0	0	0	0	0	0	
	Eucalyptus	0	0	0	0	0	1	0	0.0%
	Fir	0	0	0	0	14	0	0	100.0%
	Pine	0	2	0	0	0	10	1	76.9%
	Thyme	0	1	0	0	0	3	9	69.2%
	Missing	0	0	0	0	0	0	0	
	Overall Percent	0.0%	17.6%	0.0%	0.0%	27.5%	35.3%	19.6%	74.5%

**Table 5:** Discriminatory power of the LDA model based on the significant volatile compound according to the honey code.

Chemometric Technique	Prediction Rate	Honey Code	Predicted Group Membership					Total Honey Samples (*n* = 151)
LDA	%		CCC	E	F	P	T	
Original ^a^	Count	CCC	38	2	0	2	0	42
		E	0	4	0	0	0	4
		F	0	0	31	0	0	31
		P	0	0	0	39	0	39
		T	0	0	0	1	34	35
	%	CCC	90.5	4.8	0.0	4.8	0.0	100.0
		E	0.0	100.0	0.0	0.0	0.0	100.0
		F	0.0	0.0	100.0	0.0	0.0	100.0
		P	0.0	0.0	0.0	100.0	0.0	100.0
		T	0.0	0.0	0.0	2.9	97.1	100.0
Cross validated ^b,c^	Count	CCC	35	2	1	4	0	42
		E	1	3	0	0	0	4
		F	0	1	29	1	0	31
		P	0	0	1	37	1	39
		T	0	2	0	4	29	35
	%	CCC	83.3	4.8	2.4	9.5	0.0	100.0
		E	25.0	75.0	0.0	0.0	0.0	100.0
		F	0.0	3.2	93.5	3.2	0.0	100.0
		P	0.0	0.0	2.6	94.9	2.6	100.0
		T	0.0	5.7	0.0	11.4	82.9	100.0

^a^ 96.7% of grouped cases using the original method were correctly classified. ^b^ Cross validation was performed only for those cases in the analysis. In cross validation, each case is classified by the functions derived from all cases rather than that particular case. ^c^ 88.1% of cross validated grouped cases were correctly classified.

**Table 6:** Classification of clover, citrus, chestnut, eucalyptus, fir, pine, and thyme honeys according to the honey code using the 57 volatile compounds and *k*-NN analysis.

Partition	Observed	Predicted					
		CCC	E	F	P	T	Percent Correct
Training	CCC	26	1	0	3	0	86.7%
	E	3	1	0	0	0	25.0%
	F	0	0	25	1	0	96.2%
	P	0	0	1	27	0	96.4%
	T	6	0	0	6	11	47.8%
	Overall Percent	31.5%	1.8%	23.4%	33.3%	9.9%	81.1%
Holdout	CCC	10	0	0	2	0	83.3%
	E	0	0	0	0	0	
	F	0	0	5	0	0	100.0%
	P	0	0	0	11	0	100.0%
	T	2	0	0	4	6	50.0%
	Missing	0	0	0	0	0	
	Overall Percent	30.0%	0.0%	12.5%	42.5%	15.0%	80.0%

**Table 7:** Classification of clover, citrus, chestnut, eucalyptus, fir, pine, and thyme honeys according to the honey code using the forced predictors and, in total, 10 volatile compounds and *k*-NN analysis.

Partition	Observed	Predicted					
		CCC	E	F	P	T	Percent Correct
Training	CCC	28	0	0	2	2	87.5%
	E	2	0	0	0	1	0.0%
	F	0	0	20	0	0	100.0%
	P	0	0	1	27	1	93.1%
	T	2	0	0	1	23	88.5%
	Overall Percent	29.1%	0.0%	19.1%	27.3%	24.5%	89.1%
Holdout	CCC	5	0	1	3	1	50.0%
	E	0	0	0	0	1	0.0%
	F	0	0	8	3	0	72.7%
	P	0	0	1	8	1	80.0%
	T	2	0	0	2	5	55.6%
	Missing	0	0	0	0	0	
	Overall Percent	17.1%	0.0%	24.4%	39.0%	19.5%	63.4%

## Supplementary Materials

The following are available online at https://www.mdpi.com/2304-8158/8/10/508/s1, Table S1: Standardized canonical discriminant function coefficients of the discrimination model—structure matrix of the LDA model according to the botanical origin of honey; Table S2: Rotated component matrix of volatile compounds used for the botanical origin differentiation of clover, citrus, chestnut, eucalyptus, fir, pine, and thyme honeys; Table S3: Standardized canonical discriminant function coefficients of the discrimination model—structure matrix of the LDA model according to the honey code; Table S4: Rotated component matrix of volatile compounds used for the distinction of clover, citrus, chestnut, eucalyptus, fir, pine, and thyme honeys according to the honey code; Figure S1: A typical gas chromatogram of citrus honey (no. 4) from Spain indicating selected key volatile compounds. 10: Herboxide (isomer II). 11: Lilac aldehyde C (isomer III). 12: Dill ether. IS: internal standard; Figure S2: A typical gas chromatogram of chestnut honey (no. 2) from Portugal indicating selected key volatile compounds. 13: Octane. 14: 5-methyl-2-Furaldehyde. 15: Benzaldehyde. 16: Benzeneacetaldehyde. IS: internal standard; Figure S3: A typical gas chromatogram of eucalyptus honey (no. 1) from Portugal indicating selected key volatile compounds. 17: Heptane. 18: beta-Damascenone IS: internal standard; Figure S4: A typical gas chromatogram of fir honey (no. 6) from Greece indicating selected key volatile compounds. 19: Nonane. 20: 1-(2-furanyl)-Ethanone. 21: 6-methyl-5-Hepten-2-one. 22: 5-methyl-4-Nonene. 23: Hexanoic acid ethyl ester. 24: Heptanoic acid ethyl ester. 25: Nonanal. 26: *alpha*-Isophorone. 27: 4-Ketoisophorone. 28: 2-Hydroxyisophorone. 29: Octanoic acid ethyl ester. 30: Decane. 31: Nonanoic acid ethyl ester. 32: Geranyl acetone. IS: internal standard; Figure S5: A typical gas chromatogram of pine honey (no. 2) from Greece indicating selected key volatile compounds. 33: Acetic acid. 34: Octane. 35: *alpha*-Pinene. 36: *beta*-Thujone. IS: internal standard; Figure S6: A typical gas chromatogram of thyme pine (no. 6) from Egypt indicating selected key volatile compounds. 37: *alpha*-Pinene. 38: I-Octen-3-ol. 39: *alpha*-Terpinene. 40: *para*-Cymene. 41: Camphor. 42: Carvacrol methyl ether. 43: Thymoquinone. 44: 4,7,7-trimethylbyciclo(3.3.0)-Octan-2-one. IS: internal standard.
