# Supplementary material for: The Honey Volatile Code: A Collective Study and Extended Version
Source: Foods. 2019 Oct 17;8(10):508. doi: 10.3390/foods8100508 (PMC6835600; doi:10.3390/foods8100508)
Supplement: Supplementary file 1 [file foods-08-00508-s001.pdf]

foods

Table S1. Standardized canonical discriminant functions coefficients of the discrimination model-Structure matrix of the LDA model with respect to honey botanical origin.

Structure Matrix						
**Volatile compounds/Markers of botanical origin**	**Function**					
	**1**	**2**	**3**	**4**	**5**	**6**
**Octanoic acid ethyl ester**	**0.157 ***	0.066	−0.007	−0.011	−0.046	−0.045
**Nonanoic acid ethyl ester**	**0.125 ***	0.043	−0.034	−0.002	−0.053	−0.025
**Decanoic acid ethyl ester**	**0.123 ***	0.052	−0.034	−0.052	−0.052	0.013
**Dodecanoic acid ethyl ester**	**0.094 ***	0.040	−0.029	−0.016	−0.035	−0.001
**Decanal**	**0.082 ***	0.042	−0.028	−0.057	−0.006	0.022
**Nonanal**	**0.075 ***	0.027	−0.029	−0.031	−0.027	0.013
**5-methyl-4-Nonene**	**0.066 ***	0.019	−0.017	−0.024	−0.008	0.016
**Hexanoic acid ethyl ester**	**0.060 ***	0.023	0.014	0.001	0.008	−0.004
**Keptanoic acid ethyl ester**	**0.053 ***	0.013	−0.014	−0.006	−0.011	0.007
**4-Ketoisophorone**	**0.052 ***	0.009	−0.007	0.009	0.006	0.015
**Octanol**	**0.046 ***	0.025	−0.022	−0.025	−0.040	−0.013
**Tetradecanoic acid ethyl ester**	**0.042 ***	0.022	−0.013	−0.028	−0.007	0.011
**Geranyl acetone**	**0.041 ***	0.006	−0.009	−0.006	−0.001	0.012
**6-methyl-5-Hepten-2-one**	**0.034 ***	0.021	0.002	0.028	0.017	0.009
**1-(2-furanyl)-Ethanone**	**0.033 ***	0.015	−0.001	−0.028	−0.001	0.005
**alpha-Isophorone**	**0.028 ***	0.012	−0.008	−0.016	−0.004	0.007
**Decanol**	**0.027 ***	0.004	−0.006	−0.003	−0.003	0.006
**Hexadecanoic acid ethyl ester**	−0.019	**0.186 ***	−0.009	−0.114	−0.005	−0.027
Phenylethylalcohol	−0.009	−0.014	0.166 *	−0.113	0.080	−0.063
Formic acid ethyl ester	−0.008	−0.012	0.143 *	−0.097	0.068	−0.054
Hotrienol	−0.009	0.001	0.139 *	0.025	0.123	−0.042
Pentanoic acid	−0.008	−0.012	0.132 *	−0.088	0.055	−0.058
5-methyl-2-phenylHexenal	−0.007	−0.011	0.130 *	−0.089	0.062	−0.049
Benzeneacetonitrile	−0.007	−0.011	0.127 *	−0.086	0.061	−0.048
Thymol methyl ether[b]	−0.008	−0.005	0.123 *	−0.090	0.058	−0.049
para-Cymene	−0.007	−0.010	0.120 *	−0.082	0.061	−0.045
Borneol[b]	−0.010	−0.004	0.118 *	−0.088	0.054	−0.040
Camphor	−0.007	−0.010	0.118 *	−0.080	0.056	−0.044
4-Terpineol[b]	−0.005	−0.004	0.118 *	−0.094	0.065	−0.055
1-Octen-3-ol	−0.007	−0.010	0.117 *	−0.080	0.056	−0.044
Thymoquinone[b]	−0.010	−0.009	0.116 *	−0.081	0.064	−0.054
gamma-Terpinene	−0.006	−0.010	0.112 *	−0.077	0.054	−0.042
Thymol[b]	−0.016	0.005	0.112 *	−0.086	0.049	−0.034
Decane	−0.032	0.224	0.127	0.577 *	0.153	−0.131
alpha-Terpinolene	−0.014	0.079	0.076	−0.208 *	0.015	−0.051
Lilac aldehyde D (isomer IV)	−0.014	0.079	−0.106	−0.146 *	0.062	0.019
Benzeneacetaldehyde	0.022	0.015	0.122	−0.137 *	0.070	−0.075
2-hydroxyIsophorone	0.046	0.017	0.008	0.065 *	0.032	0.013
2-ethyl-1-Hexanol	0.060	0.018	−0.012	0.064 *	−0.034	−0.030
Undecanoic acid ethyl ester	0.044	0.038	−0.019	−0.055 *	−0.014	0.009
3,4,5-trimethylPhenol	0.031	0.023	−0.012	−0.033*	−0.008	0.007
Dill ether	−0.023	−0.025	−0.265	−0.064	0.364 *	0.102
Lilac aldehyde C (isomer III)	−0.026	0.002	−0.289	−0.103	0.362 *	0.105
alpha, 4-dimethyl-3-cyclohexene-1-acetaldehyde	−0.019	−0.021	−0.210	−0.055	0.298 *	0.081
Herboxide (isomer II)	−0.019	−0.004	−0.205	−0.068	0.266 *	0.076
Lilac aldehyde B (isomer II)	−0.016	−0.015	−0.192	−0.027	0.252 *	0.075
Lilac aldehyde A (isomer I)	−0.014	−0.015	−0.158	−0.041	0.222 *	0.061
Methylanthranilate	−0.012	−0.013	−0.164	−0.026	0.197 *	0.063
Dodecanoic acid	−0.009	−0.008	−0.039	0.067	−0.185 *	−0.148
cis-Linalool oxide	0.035	0.046	−0.042	−0.066	0.143 *	0.022

Nonanol	−0.011	0.036	−0.035	−0.003	−0.141 *	−0.110
Furfural	0.051	0.058	−0.058	−0.071	−0.087 *	0.017
Octanal	0.043	0.025	−0.022	−0.008	−0.075 *	−0.012
Nonane	0.032	0.011	−0.023	0.018	−0.074 *	−0.049
Tridecanoic acid ethyl ester	0.006	0.042	−0.024	−0.052	−0.064 *	−0.039
Acetic acid ethyl ester	−0.010	−0.005	0.026	0.023	0.137	0.545 *
2-methyl-1-Butanol	−0.008	−0.005	0.060	0.000	−0.105	0.395 *
3-methylbutanal	−0.010	−0.008	0.074	−0.017	−0.083	0.386 *
Heptane	−0.032	0.069	0.236	0.140	0.157	0.369 *
2-methylbutanal	−0.009	−0.010	0.069	−0.048	0.012	0.158 *
Eugenol[b]	−0.035	0.050	0.107	−0.056	0.007	0.146 *
Benzaldehyde	0.035	0.032	0.023	−0.046	−0.008	−0.055 *

Pooled within-groups correlations between discriminating variables and standardized canonical discriminant functions. Variables ordered by absolute size of correlation within function. *. Largest absolute correlation between each variable and any discriminant function. b. This variable not used in the analysis-Tolerance test.

Table S2. Rotated component matrix of volatile compounds used for the botanical origin differentiation of clover, citrus, chestnut, eucalyptus, fir, pine, and thyme honeys.

Rotated Component Matrix[a]	Component															
	1	2	3	4	5	6	7	8	9	10	11	12	13	14	15	16
1-Decanol	0.921											0.206				−0.124
3,4,5-trimethylPhenol	0.898			0.213	0.118			0.125				0.165				−0.156
Heptanoic acid ethyl ester	0.847	0.165			0.106										−0.169	0.294
Octanol	0.797	0.195		0.354	0.111			0.166				0.112		0.231	0.125	
**Hexanoic acid ethyl ester**	0.751	0.383													−0.156	**0.347**
Benzaldehyde	0.565	0.402			0.565					0.243						−0.135
**Undecanoic acid ethyl ester**		**0.907**						0.236				−0.110				
6-methyl-5-Hepten-2-one		0.880						−0.135			0.122	−0.123				
Geranyl acetone		0.841												−0.149		−0.110
Decanoic acid ethyl ester	0.321	0.790		0.229	0.148							0.105		0.164	−0.117	0.183
Octanoic acid ethyl ester	0.302	0.727		0.343	0.140	−0.119	−0.119			−0.102		0.152		0.212	−0.106	0.270
Tetradecanoic acid ethyl ester		0.705						0.249				0.391		−0.162		
Nonanoic acid ethyl ester	0.168	0.689		0.456	0.128		−0.104							0.240		0.159
Dodecanoic acid ethyl ester	0.526	0.544			0.175							0.309		0.158	−0.170	0.301
**para-Cymene**			**0.988**													
gamma-Terpinene			0.978													
1-Octen-3-ol			0.974												0.120	
(1*R*, 4*S*)-1,7,7-trimethyl-bicyclo-(2.2.1)-Heptan-2-one			0.966												0.138	
**2-hydroxyIsophorone**				**0.923**				0.110								0.111
Nonanal		0.297		0.862	0.195									0.114		
alpha-Isophorone				0.838												
4-Ketoisophorone	0.552			0.770	0.110											
Decanal	0.190	0.493		0.727	0.240											
**Nonane**				0.123	**0.918**											
Octanal	0.266	0.152		0.279	0.839							0.287				
Furfural	0.235	0.215		0.163	0.779			0.372								
**Dill ether**						**0.906**	0.196									
alpha,4-dimethyl-3-cyclohexene-1-Acetaldehyde						0.885	0.224									
Herboxide (isomer II)						0.860		0.132								
**Lilac aldehyde C(isomer III)**						0.232	**0.889**	0.159								
Methylanthranilate							0.788									
Lilac aldehyde B (isomer II)							0.711									
Lilac aldehyde A (isomer I)						0.422	0.575						0.102			
**Lilac aldehyde D (isomer IV)**					0.129	0.205		**0.794**				−0.121				−0.132
Tridecanoic acid ethyl ester						−0.110		0.730				0.148		0.139		0.238
alpha-Terpinolene			0.533					0.603			0.208				−0.189	−0.123
cis-Linalool oxide		0.312		0.311		0.267		0.583						−0.156		
**Acetic acid ethyl ester**									**0.921**							
2-methyl-1-Butanol									0.898	0.103						
3-methylButanal									0.670						0.222	
**5-methyl-2-phenylHexenal**										**0.776**						
Formic acid ethyl ester										0.759						0.100
Benzeneacetaldehyde	0.282	0.192			0.318					0.737		0.259	0.169			
Phenylethylalcohol										0.495			0.453			−0.130
**Decane**											**0.860**					
Hexadecanoic acid ethyl ester											0.854					
Heptane		−0.101	0.343						0.167	−0.126	0.580			−0.190	0.412	0.131
**1-(2-furanyl)-Ethanone**	0.602				0.220							**0.699**				
5-methyl-4-Nonene	0.553	0.152			0.229							0.691				
2-ethyl-1-Hexanol	0.440	0.304		0.216	0.283							0.601		0.304		0.117
**Benzeneacetonitrile**													**0.923**			
Pentanoic acid										0.118			0.793			0.129
**Nonanol**					0.130			0.329			0.102			**0.777**	0.163	
Hotrienol								0.106	−0.103		0.206			−0.477	0.211	0.165
**2-methylButanal**			0.262												**0.776**	0.108
Dodecanoic acid							−0.121			−0.152			−0.108	0.128	−0.167	−0.607

Extraction Method: Principal Component Analysis. Rotation Method: Varimax with Kaiser Normalization. a. Rotation converged in 8 iterations.

Table S3. Standardized canonical discriminant functions coefficients of the discrimination model-Structure matrix of the LDA model with respect to honey code.

Structure Matrix				
**Volatile compounds/ Markers of the honey code**	**Function**			
	**1**	**2**	**3**	**4**
**Octanoic acid ethyl ester**	**0.204** *	−0.028	0.005	0.026
**Nonanoic acid ethyl ester**	**0.166** *	−0.040	−0.025	0.006
**Decanoic acid ethyl ester**	**0.161** *	−0.031	−0.022	−0.052
**Dodecanoic acid ethyl ester**	**0.126** *	−0.034	−0.010	−0.018
**Decanal**	**0.109** *	−0.021	0.006	−0.070
**5-methyl-4-Nonene**	**0.088** *	−0.012	−0.004	−0.039
**Hexanoic acid ethyl ester**	**0.081** *	−0.001	0.028	0.009
**2-ethyl-1-hexanol**	**0.080** *	−0.039	−0.003	0.073
**Heptanoic acid ethyl ester**	**0.071** *	−0.013	−0.006	−0.015
**4-Ketoisophorone**	**0.069** *	−0.011	0.010	−0.010
**Furfural**	**0.067** *	−0.054	−0.042	−0.059
**2-hydroxyIsophoronr**	**0.062** *	−0.028	0.059	0.044
**1-Octanol**	**0.062** *	−0.019	−0.025	−0.013
**Undecanoic acid ethyl ester**	**0.059** *	−0.016	0.003	−0.050
**Octanal**	**0.058** *	−0.028	−0.042	0.014
**Tetradecanoic acid ethyl ester**	**0.057** *	−0.011	0.002	−0.033
**Geranyl acetone**	**0.055** *	−0.005	−0.003	−0.020
**6-methyl-5-Hepten-2-one**	**0.047** *	−0.022	0.040	0.018
**Benzaldehyde**	**0.047** *	0.020	0.010	0.012
**1-(2-furanyl)-Ethanone**	**0.044** *	0.003	0.004	−0.026
**3,4,5-trimethylPhenol**	**0.042** *	−0.010	0.001	−0.032
**alpha-Isophorone**	**0.037** *	−0.006	0.000	−0.021
**1-Decanol**	**0.036** *	−0.004	−0.004	−0.010
**Phenylethylalcohol**	−0.013	**0.181** *	0.067	0.011
**Formic acid ethyl ester**	−0.011	**0.155** *	0.057	0.010
Thymol[b]	−0.015	**0.149** *	0.047	−0.001
Thymoquinone[b]	−0.016	**0.144** *	0.051	−0.001
**Pentanoic acid**	−0.011	**0.144** *	0.045	0.018
**5-methyl-2-phenylHexenal**	−0.010	**0.141** *	0.052	0.009
**Benzeneacetaldehyde**	0.029	**0.140** *	0.057	−0.022
**Borneol**	−0.010	**0.139** *	0.052	0.007
**Benzeneacetonitrile**	−0.010	**0.138** *	0.051	0.009
2-methoxy-4-methyl-1-(methylethylBenzene) [b]	−0.012	**0.134** *	0.047	0.003
(1*R*, 4*S*) -1,7,7-trimethyl-bicyclo-(2.2.1)-Heptan-2-one [b]	−0.011	**0.134** *	0.044	0.003
4-Terpineol[b]	−0.005	**0.132** *	0.053	0.009
***para*-Cymene**	−0.010	**0.130** *	0.050	0.006
**Eugenol**	−0.009	**0.128** *	0.047	0.008
**1-Octen-3-ol**	−0.009	**0.128** *	0.047	0.008
Carvacrol methyl ether[b]	−0.010	**0.124** *	0.032	−0.002
**gamma-Terpinene**	−0.009	**0.122** *	0.045	0.008
**Undecane**	−0.009	**0.120** *	0.044	0.007
**dl-limonene**	0.008	**0.115** *	0.042	0.001
**3-hydroxy-4-phenyl-2-Butanone**	−0.008	**0.112** *	0.041	0.007
**4,7,7-trimethylbyciclo(3.3.0)Octan-2-one**	−0.008	**0.106** *	0.039	0.007
**Sabinene**	−0.008	**0.106** *	0.039	0.007
***alpha*-Terpinene**	−0.008	**0.105** *	0.039	0.007
Linalool	0.001	0.099 *	0.083	0.024
Heptane	−0.034	0.005	0.364 *	0.066
Hotrienol	−0.012	0.094	0.145 *	0.093
Nonane	0.043	−0.025	−0.057 *	0.049
Decane	−0.020	−0.215	0.392	0.486 *
Lilac aldehyde C	−0.032	−0.136	0.095	−0.412 *

Dill ether	−0.030	−0.114	0.089	−0.370 *
alpha,4-dimethyl-3-cyclohexene-1-Acetaldehyde	−0.025	−0.091	0.076	−0.312 *
Herboxide (isomer II)	−0.024	−0.098	0.072	−0.307 *
Lilac aldehyde B	−0.021	−0.098	0.069	−0.264 *
Lilac aldehyde A	−0.020	−0.071	0.059	−0.240 *
Methylanthranilate	−0.017	−0.085	0.048	−0.225 *
Lilac aldehyde D	−0.013	−0.065	0.037	−0.174 *
Dodecanoic acid	−0.013	−0.039	−.158	0.167 *
Acetic acid ethyl ester	−0.011	−0.057	0.032	−0.151 *
beta-Thujone	−0.010	−0.031	−0.124	0.130 *
cis-Linalool oxide	0.047	−0.027	0.102	−0.129 *
Nonanoic acid	−0.009	−0.028	−0.111	0.117 *
Decanoic acid	−0.009	−0.027	−0.108	0.114 *

Pooled within-groups correlations between discriminating variables and standardized canonical discriminant functions. Variables ordered by absolute size of correlation within function. * Largest absolute correlation between each variable and any discriminant function. b. This variable not used in the analysis-Tolerance test.

Table S4. Rotated component matrix of volatile compounds used for the distinction of clover, citrus, chestnut, eucalyptus, fir, pine, and thyme honeys according to honey code.

Rotated Component Matrix[a]															
	**Component**														
	**1**	**2**	**3**	**4**	**5**	**6**	**7**	**8**	**9**	**10**	**11**	**12**	**13**	**14**	**15**
Formic acid ethyl ester								0.705						0.327	
Acetic acid ethyl ester															−0.901
Heptane	0.350												−0.714		−0.253
Furfural		0.244	0.266	0.198	0.671		0.189				0.427		0.149		
Pentanoic acid								0.155				0.868			
Nonane			0.151	0.159	0.912		0.121								
Hexanoic acid ethyl ester		0.394	0.791						−0.123	−0.115	−0.136			−0.112	0.150
Benzaldehyde		0.405	0.602		0.528			0.179			0.113			0.184	
Octanal		0.186	0.278	0.318	0.769		0.382								
Benzeneacetaldehyde		0.200	0.266		0.232	−0.112	0.364	0.599			0.148			0.374	
5-methyl-4-Nonene		0.162	0.484				0.782						0.105		
Decane													−0.769		
dl-Limonene	0.893														
1-Octen-3-ol	0.988														
2-ethyl-1-Hexanol		0.359	0.408	0.245	0.255		0.630				−0.121				
**1-(2-furanyl)-Ethanone**			0.524				**0.809**								
cis-Linalool oxide		0.311		0.301		0.268					0.589				
alpha-Terpinene	0.530														
Heptanoic acid ethyl ester		0.167	0.877						−0.111	−0.100			0.117		0.135
Octanol		0.198	0.778	0.373			0.198				0.151				
**para-Cymene**	**0.989**														
Linalool	0.968	0.138													
Nonanal		0.312		0.874									0.121		
Octanoic acid ethyl ester		0.756	0.316	0.352		−0.117	0.167		−0.132	−0.118			0.119	−0.124	0.157
**Lilac aldehyde B (isomer II)**									**0.860**						
Hotrienol													−0.497		0.157
Herboxide (isomer II)						0.827					0.180				
**Undecane**								**0.849**							
Decanal		0.490	0.196	0.738							0.137		0.108		
Nonanoic acid					0.960										
Sabinene	0.951														
beta-Thujone										0.870					
Phenylethylalcohol								0.267				0.236		0.660	
gamma-Terpinene	0.973														
**Benzeneacetonitrile**												**0.870**		0.241	
6-methyl-5-Hepten-2-one		0.881					−0.135				−0.141				
alpha-Isophorone				0.835											
**2-hydroxyIsophorone**				**0.925**											
**Decanol**			**0.884**				0.287								
Nonanoic acid ethyl ester		0.699	0.185	0.459		−0.109			−0.112	−0.109			0.155		0.133
3,4,5-trimethylPhenol			0.861	0.226			0.273				0.157				
**Decanoic acid**					**0.965**					0.107					
Borneol	0.978														
**Decanoic acid ethyl ester**		0.798	0.330	0.234			0.141		−0.109	−0.109			**0.158**		0.131
**Dodecanoic acid**										**0.875**					
**Dodecanoic acid ethyl ester**		0.572	0.523				0.359		−0.125	−0.124			0.155	−0.101	**0.173**
Tetradecanoic acid ethyl ester		0.711					0.327				0.195				
**Undecanoic acid ethyl ester**		**0.892**	0.109								0.294				
4-Ketoisophorone			0.527	0.776			0.151								
5-methyl-2-phenylHexenal								0.823							
Lilac aldehyde A (isomer I)						0.484			0.516			0.151		−0.100	
**4,7,7-trimethylbyciclo (3.3.0)-Octan-2-one**														**0.839**	
Lilac aldehyde C (isomer III)						0.303			0.810		0.153				
Geranyl acetone		0.827													
**Lilac aldehyde D (isomer IV)**						0.120					**0.882**				
**Dill ether**						**0.923**			0.128						
alpha-4-dimethyl-3-cyclohexene-1-Acetaldehyde						0.906			0.139						

Extraction Method: Principal Component Analysis. Rotation Method: Varimax with Kaiser Normalization. a. Rotation converged in 7 iterations.

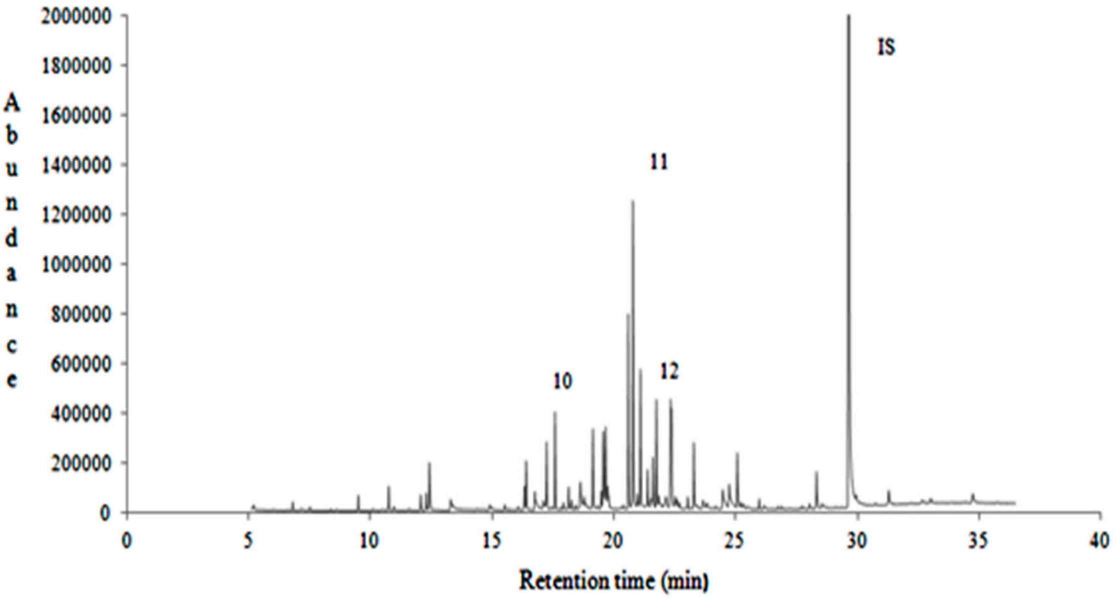

Figure S1. A typical gas chromatogram of citrus honey (no. 4) from Spain indicating selected key volatile compounds. 10: Herboxide (isomer II). 11: Lilac aldehyde C (isomer III). 12: Dill ether. IS: internal standard.

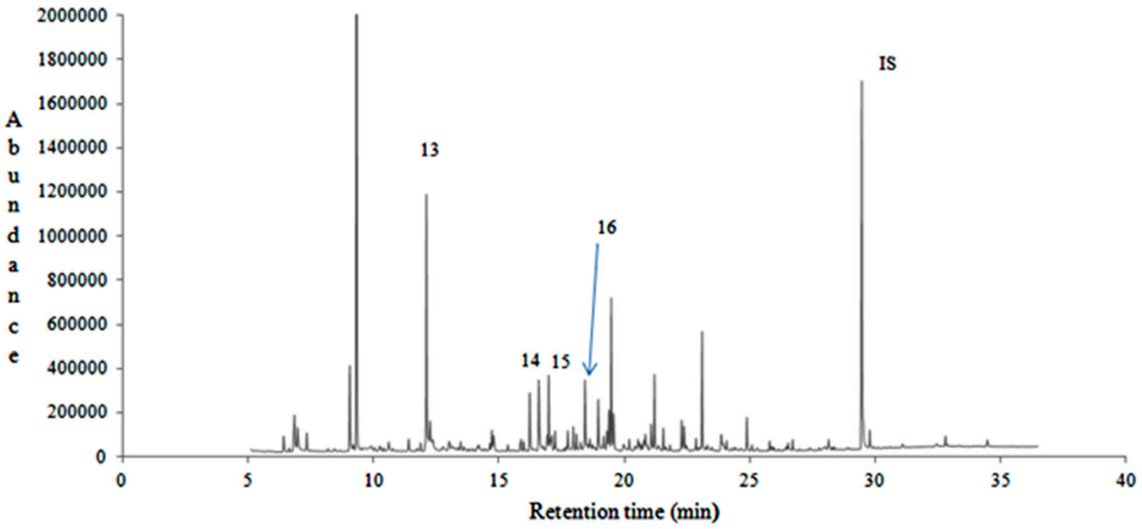

Figure S2. A typical gas chromatogram of chestnut honey (no. 2) from Portugal indicating selected key volatile compounds. 13: Octane. 14: Furfural. 15: Benzaldehyde. 16: Benzeneacetaldehyde. IS: internal standard.

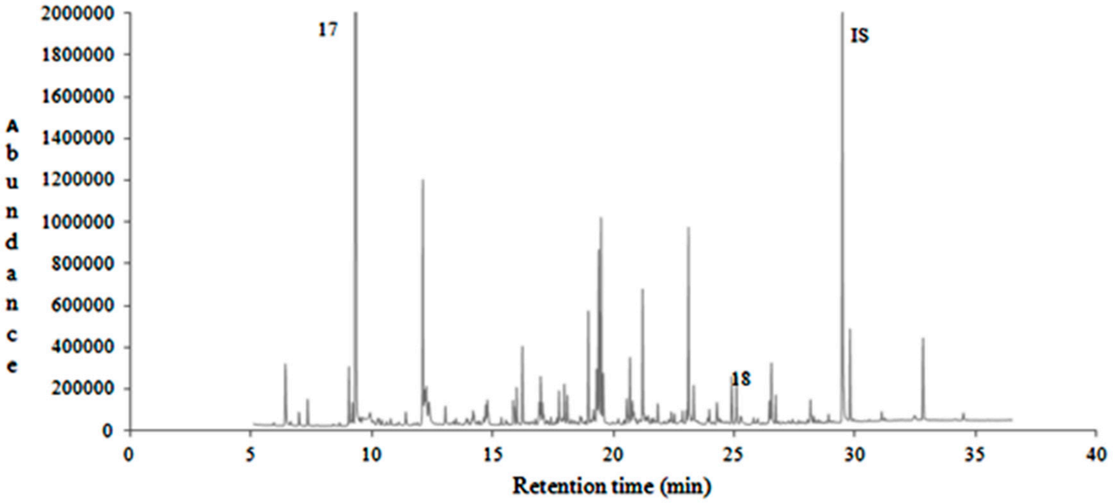

Figure S3. A typical gas chromatogram of eucalyptus honey (no. 1) from Portugal indicating selected key volatile compounds. 17: Heptane. 18: beta-Damascenone IS: internal standard.

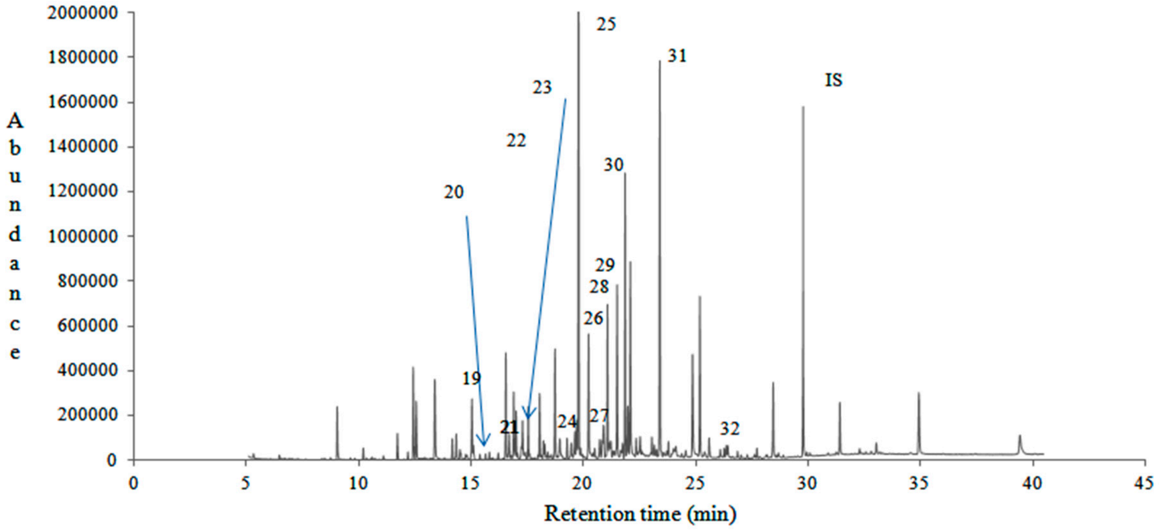

Figure S4. A typical gas chromatogram of fir honey (no. 6) from Greece indicating selected key volatile compounds. 19: Nonane. 20: 1-(2-furanyl)-Ethanone. 21: 6-methyl-5-Hepten-2-one. 22: 5-methyl-4-Nonene. 23: Hexanoic acid ethyl ester. 24: Heptanoic acid ethyl ester. 25: Nonanal. 26: alpha-Isophorone. 27: 4-Ketoisophorone. 28: 2-Hydroxyisophorone. 29: Octanoic acid ethyl ester. 30: Decane. 31: Nonanoic acid ethyl ester. 32: Geranyl acetone. IS: internal standard.

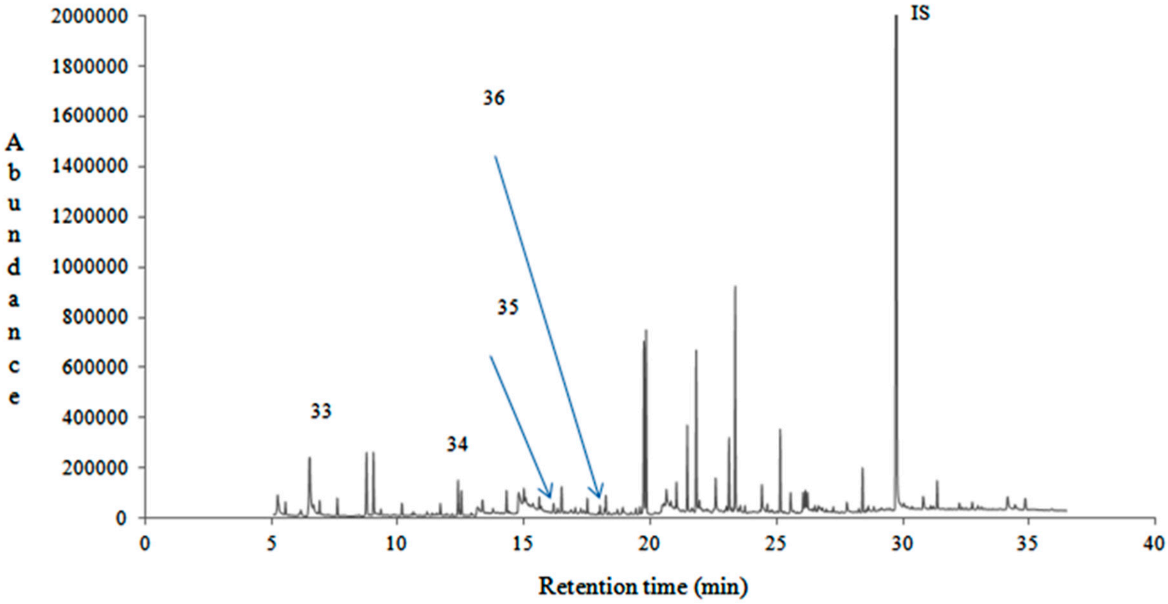

Figure S5. A typical gas chromatogram of pine honey (no. 2) from Greece indicating selected key volatile compounds. 33: Acetic acid. 34: Octane. 35: alpha-Pinene. 36: beta-Thujone. IS: internal standard.

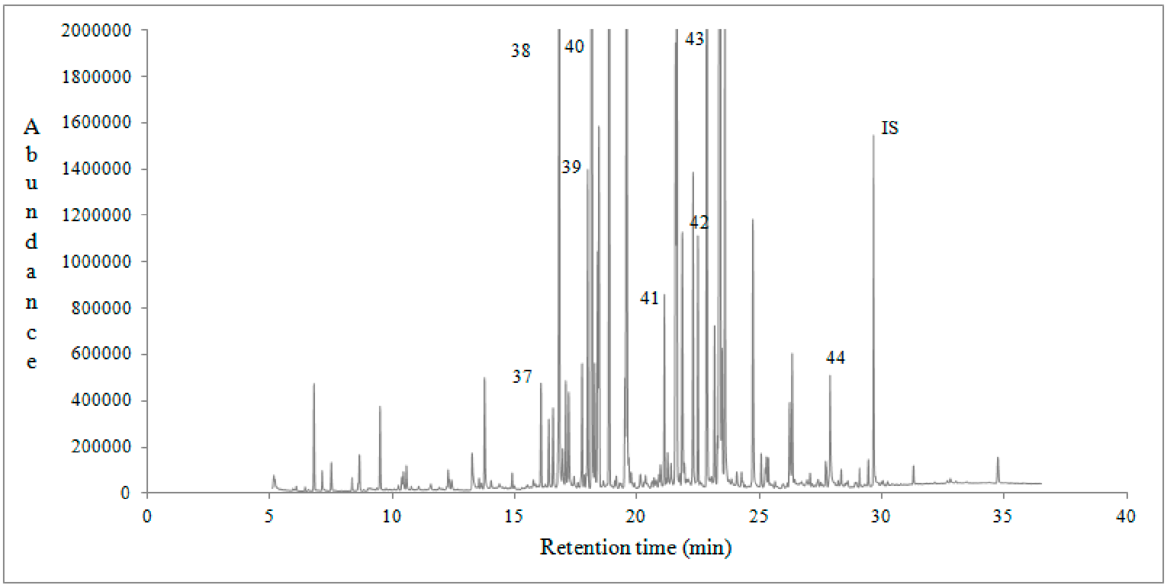

Figure S6. A typical gas chromatogram of thyme pine (no. 6) from Egypt indicating selected key volatile compounds. 37: alpha-Pinene. 38: I-Octen-3-ol. 39: alpha-Terpinene. 40: para-Cymene. 41: Camphor. 42: Carvacrol methyl ether. 43: Thymoquinone. 44: 4,7,7-trimethylbyciclo[3.3.0)-Octan-2-one. IS: internal standard.
